# Periodic dietary restriction of animal products induces metabolic reprogramming in humans with effects on cardiometabolic health

**DOI:** 10.1038/s44324-025-00057-2

**Published:** 2025-04-09

**Authors:** Konstantinos Rouskas, Ozvan Bocher, Alexandros Simistiras, Christina Emmanouil, Panagiotis Mantas, Anargyros Skoulakis, Young-Chan Park, Alexandros Dimopoulos, Stavros Glentis, Gabi Kastenmüller, Eleftheria Zeggini, Antigone S. Dimas

**Affiliations:** 1 Institute for Bioinnovation, Biomedical Sciences Research Center ‘Alexander Fleming’, Fleming 34, 16672 Vari, Greece; 2 Institute of Applied Biosciences, Centre for Research & Technology Hellas, Thessaloniki, Greece; 3 Institute of Translational Genomics, Helmholtz Zentrum München – German Research Center for Environmental Health, Neuherberg, Germany; 4 Institute of Computational Biology, Helmholtz Zentrum München – German Research Center for Environmental Health, Neuherberg, Germany; 5 German Center for Diabetes Research (DZD), Neuherberg, Germany; 6 Technical University of Munich (TUM) and Klinikum Rechts der Isar, TUM School of Medicine and Health, Munich, Germany

**Keywords:** Metabolism, Metabolomics, Endocrine system and metabolic diseases

## Abstract

Dietary interventions constitute powerful approaches for disease prevention and treatment. However, the molecular mechanisms through which diet affects health remain underexplored in humans. Here, we compare plasma metabolomic and proteomic profiles between dietary states for a unique group of individuals who alternate between omnivory and restriction of animal products for religious reasons. We find that short-term restriction drives reductions in levels of lipid classes and of branched-chain amino acids, not detected in a control group of individuals, and results in metabolic profiles associated with decreased risk for all-cause mortality. We show that 23% of proteins whose levels are affected by dietary restriction are druggable targets and reveal that pro-longevity hormone FGF21 and seven additional proteins (FOLR2, SUMF2, HAVCR1, PLA2G1B, OXT, SPP1, HPGDS) display the greatest magnitude of change. Through Mendelian randomization we demonstrate potentially causal effects of FGF21 and HAVCR1 on risk for type 2 diabetes, of HPGDS on BMI, and of OXT on risk for lacunar stroke. Collectively, we find that restriction-associated reprogramming improves metabolic health and emphasise high-value targets for pharmacological intervention.

## Introduction

Dietary interventions that involve the restriction of energy or of particular nutrients, without malnutrition, have been shown to delay aging and extend healthspan and lifespan in diverse species^1–5^. Although the type of restriction may vary (e.g. restriction of the level, type and timing of food consumption), studies have highlighted partially overlapping biological mechanisms of action, in evolutionarily conserved signalling pathways, as mediators of the effects of dietary restriction^1–5^. These pathways converge on key nodes of regulatory networks controlling aging-related processes and include mTOR, a protein a kinase acting in two complexes (mTORC1 and mTORC2) that mediates signalling in response to nutrient intake.

In humans, dietary restriction has been shown to have a protective effect against ageing-related disorders, including prevention of obesity and diabetes, cardiovascular disease, kidney disease, autoimmune and inflammatory conditions, and cancer^2–4^. The last decades have seen an emergence of studies addressing the impact of patterns of dietary restriction on health, with plant-based diets in particular, having attracted a lot of interest^6–8^. Plant-based diets involve the restriction of animal product consumption, and practicing individuals typically present with lower BMI, lower serum LDL levels, and lower blood pressure^8^. Greater adherence to these diets has been linked to a reduced risk of cardiovascular disease, dyslipidemia, diabetes and certain types of cancer^8,9^, but also to some health risks, including lower bone mineral density and higher risk of stroke^6^. To date, few studies have addressed the molecular mechanisms underlying the effects of plant-based diets on health and disease, but work in this direction is emerging. Recently, a study comparing vegan versus ketogenic dietary interventions demonstrated effects on the innate and adaptive immune responses respectively^9^. Another study assessed plasma proteome differences in individuals from six dietary groups and revealed that individuals consuming plant-based diets displayed different profiles of proteins linked to gastrointestinal tract and kidney function^7^. A better understanding of the molecular effects of plant-based diets will enable us to harness their positive effects on health and to minimize possible detrimental consequences.

Here we have conducted molecular profiling for the first time, of a unique group of individuals from Greece who alternate between omnivory and restriction of animal products for religious reasons. The high consistency of the dietary pattern adhered to and the predictability in switching between omnivory and restriction, renders this study similar to dietary intervention studies. We compared plasma metabolomic and proteomic profiles of practicing individuals between dietary states, and to profiles obtained in parallel from a control group of continuously omnivorous individuals and demonstrate extensive metabolic reprogramming upon animal product restriction. We suggest that this dietary pattern can be harnessed to improve health and inform the development of interventions to prevent and treat disease, but a better grasp of underlying mechanisms is necessary to minimize possible negative effects.

## Results

### Population sample description and study design

**Fig. 1:**
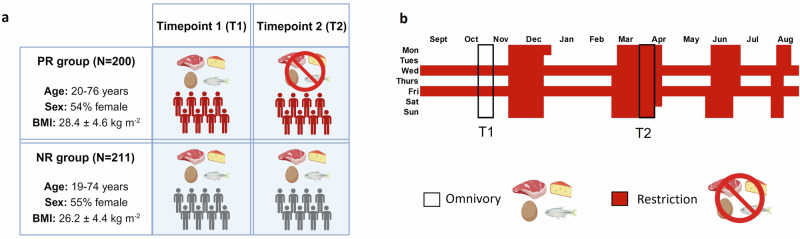
Study design and periods of animal product restriction. **a** Schematic of study design. Periodically restricted (PR) individuals alternate between omnivory and animal product restriction for religious reasons. These two dietary states were profiled at timepoint 1 (T1) and timepoint 2 (T2) respectively. Non-restricted (NR) individuals are continuously omnivorous and were also profiled at T1 and T2. Created with BioRender.com. **b** PR individuals practice animal product restriction for 180-200 days annually. Restriction is practiced during four extended periods throughout the year and on Wednesdays and Fridays of each week.

Following screening of over 1000 candidate participants, we recruited 411 apparently healthy individuals from Thessaloniki, Greece for the FastBio (Religious Fasting Biology) study (Fig. 1a, Supplementary Text S1). Participants belonged to one of two dietary groups: individuals who had undergone periodic restriction of animal products for religious reasons, for at least ten years, and individuals who were continuously omnivorous. Periodically restricted (PR) individuals (*N* = 200, age: 20–76 years, sex: 54% female, BMI: 28.4 ± 4.6 Kg m^-2^) voluntarily alternate between restriction and omnivory following the dietary regimen of the Greek Orthodox Church (Supplementary Text S2). This involves abstinence from meat, fish, dairy products and eggs (but not molluscs and shellfish) for 180-200 days annually, in a highly consistent and temporally structured manner. Restriction is practiced during four extended periods throughout the year, as well as on Wednesdays and Fridays of each week (Fig. 1b). Non-restricted (NR), control individuals (*N* = 211, age: 19–74 years, sex: 55% female, BMI: 26.2 ± 4.4 Kg m^-2^) were continuously omnivorous and did not practice any type of dietary restriction. All participants were from families living above the line of poverty. Both groups consisted mostly of married individuals (PR: 73.5%, NR: 65.9%), with tertiary education (PR: 67%, NR: 75.4%), and originated mostly from Northern Greece (PR: 68.5%, NR: 65.4%) (Supplementary Data 1). Participants were profiled at two timepoints: T1 in autumn, covering a period of omnivory for both dietary groups, and T2 in early spring, covering a three-to-four-week period of restriction for PR individuals, during Lent (Fig. 1b). Typically, during this period, PR individuals undergo restriction of protein (as a proportion of total energy intake), driven chiefly by abstinence from almost all sources of animal protein, which is not accompanied by a decrease in total energy intake (Supplementary Text S2)^10,11^.

### Animal product restriction is associated with a prominent shift in metabolomic profiles

**Fig. 2:**
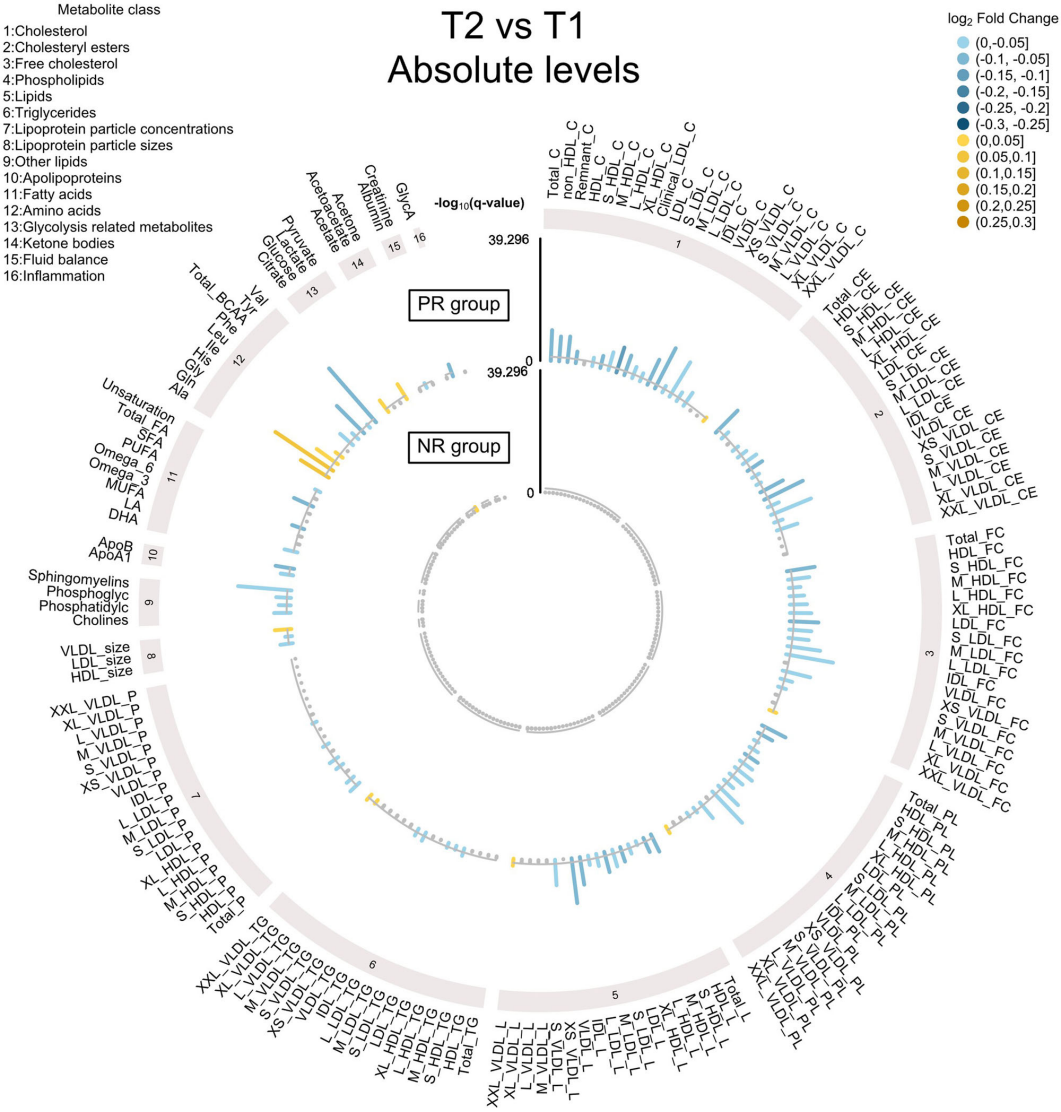
Differentially abundant metabolites (absolute levels) detected from T1 to T2 for each dietary group. Metabolites are grouped into classes. Within lipid classes 1-7, lipoproteins are grouped by type and are ordered by size. Changes in metabolite profiles are shown in the outer circle for PR individuals and in the inner circle for NR individuals. The -log_10_ of the FDR-adjusted p-value (q-value) is represented on the y-axis. Yellow bars represent an increase in metabolite levels from T1 to T2 whereas blue bars represent a decrease. Metabolites shown in grey are not significant.

**Fig. 3:**
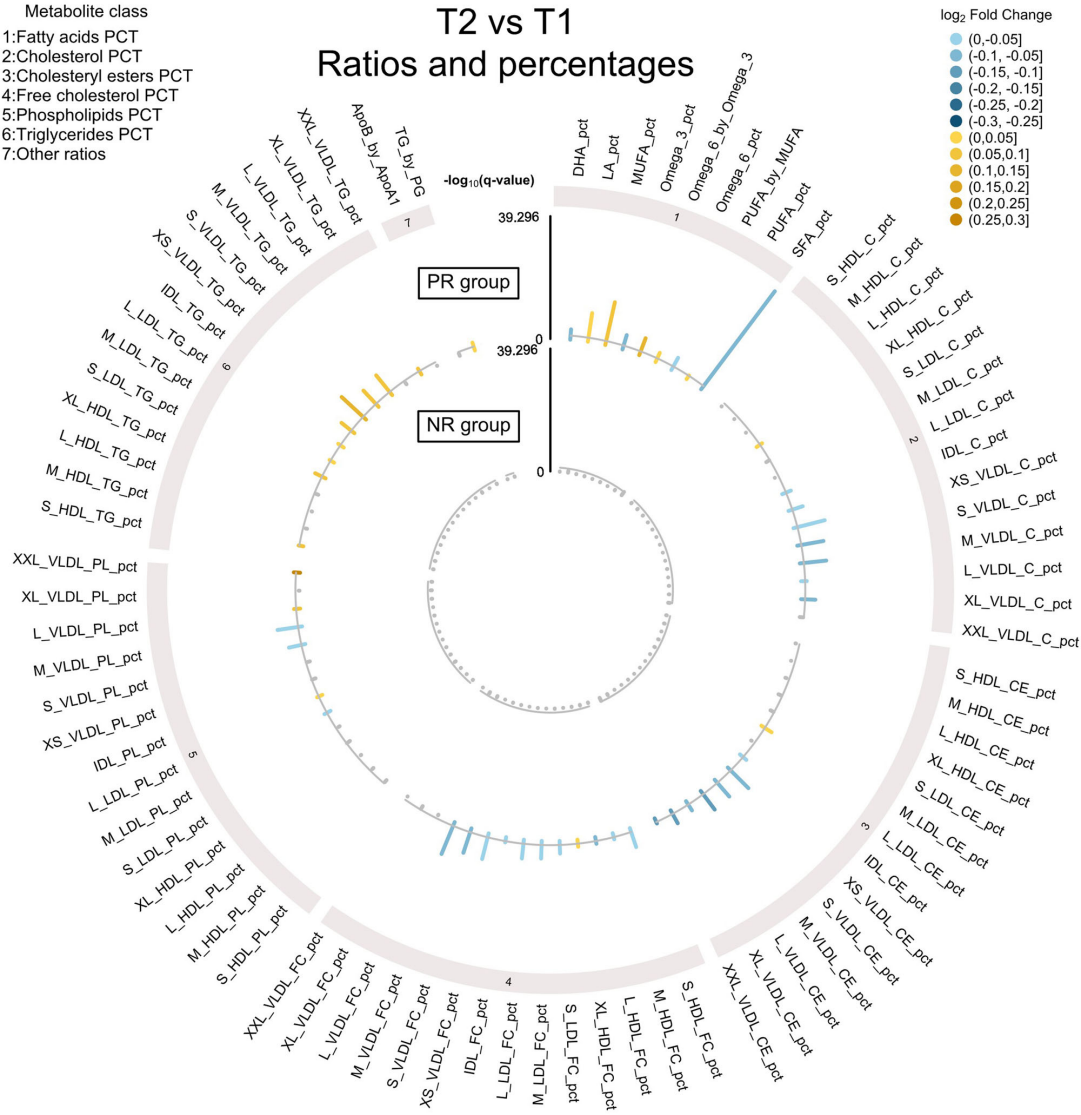
Differentially abundant metabolites (ratios and percentages) detected from T1 to T2 for each dietary group. Metabolites ratios and percentages are grouped into classes. Within each class, lipoproteins are grouped by type and are ordered by size. Changes in metabolite profiles are shown in the outer circle for PR individuals and in the inner circle for NR individuals. The -log_10_ of the FDR-adjusted p-value (q-value) is represented on the y-axis. Yellow bars represent an increase in metabolite levels from T1 to T2 whereas blue bars represent a decrease. Metabolites shown in grey are not significant.

We first sought to investigate how restriction of animal products affects plasma metabolite levels through quantification of 249 metabolites. Following quality control (QC) (Supplementary Fig. S1, Supplementary Fig. S2), we analysed 248 metabolites from 777 samples (193 PR at T1 and 190 PR at T2; 202 NR at T1 and 192 NR at T2). We found a prominent shift in metabolite profiles for the PR group with over two thirds (*N* = 166) of quantified metabolites detected at altered levels upon restriction (Fig. 2, Fig. 3, Supplementary Data 1). In the NR group, only pyruvate was detected at slightly altered levels (Fig. 2, Supplementary Data 2). The metabolite panel used in this study is enriched for lipid-related metabolites which could not directly be mapped to metabolites present in databases such as MetaboAnalyst. Although this prevented the use of such tools for interpretation of our findings, we describe changes in various metabolite classes. Most changes associated with animal product restriction (132 out of 166) involved decreased metabolite levels with reductions in multiple lipid classes including cholesterol, cholesterol esters, free cholesterol, phospholipids, and lipids from most lipoprotein particle types (HDL, IDL, LDL) and sizes. Decreased levels were also found for sphingomyelins, for the ratio of saturated to total fatty acids (SFA %) and for both ApoA1 and ApoB. Lower levels of ApoB, which is a major constituent of LDL-related lipids, are associated with decreased risk of cardiovascular events^12^, while HDL-related lipids, and its major constituent ApoA1, both have cardioprotective effects^13,14^. Reductions in levels of LDL-related lipids were of a greater magnitude compared to those recorded for HDL-related lipids. Similar patterns have been revealed in studies of individuals following a vegan diet^15,16^, a dietary pattern in close proximity to animal product restriction, and likely reflect the overall lower intake of animal fat. Furthermore, although we did not detect changes in triglyceride levels, we identify a striking pattern of increased proportions of triglycerides in VLDL particles (Fig. 3), pointing towards an increased rate of triglyceride removal from the blood to the liver.

We also found altered levels for eight out of the nine amino acids quantified upon restriction (Fig. 2), with a ~ 7% decrease detected for absolute concentrations of total BCAAs, driven by decreases in valine (9%) and leucine (5%), but not isoleucine. BCAAs, which are strong agonists of mTORC1, make up ~20% of amino acids in meat, fish, eggs and nuts^17^ and elevated levels in humans have been linked to poor health outcomes including obesity, type 2 diabetes and cardiovascular disease^18–20^. BCAA restriction on the other hand has been linked to reduced insulin resistance^21^ and to increased levels of potent metabolic regulator and pro-longevity hormone FGF21 in some^22^, but not all studies^23^. Furthermore, although protein restriction has been linked to decreased fasting blood glucose levels in overweight or obese males^24^, we did not find changes in glucose levels in the present study, which comprises participants from both sexes who span the range of BMI.

Given the similarity of the dietary pattern under study to a vegan diet, we compared our findings to those from a cross-sectional study investigating profiles of 207 metabolites, also tested in the present study, in male vegans and meat-eaters^25^, and replicated 80% of reported associations (Supplementary Fig. S3). This suggests that a short course of animal product restriction results in a metabolite profile that shares similarity to profiles of permanent vegans. Additionally, we found 58 previously unreported associations for metabolites including small VLDL, medium LDL, and large HDL classes, with decreases in cholesterol-associated levels upon restriction and identified 27 new associations for metabolites not analysed in the above study, including total BCAAs, pyruvate and LDL- and medium HDL-related lipids (Supplementary Fig. S3).

We also examined differences of metabolite profiles between dietary groups for each timepoint and found that when both groups are omnivorous, they share identical profiles (Supplementary Text S3, Supplementary Fig. S4, Supplementary Fig. S5). Upon dietary restriction however, we detected differences in the levels of 86 metabolites, of which 83 (97%) were also detected in the PR group from T1 to T2, supporting the idea that these changes are directly linked to animal product restriction. Our findings suggest that metabolomic reprogramming is rapid, but also likely transient.

Finally, to explore the effect of blood pressure on metabolite levels, we conducted a sensitivity analysis in which we adjusted further for systolic and diastolic blood pressure and found that the impact of this adjustment on our findings was minimal (Supplementary Fig. S6).

### Animal product restriction results in metabolomic profiles associated with reduced risk for all-cause mortality and with beneficial effects against cardiometabolic diseases

**Fig. 4:**
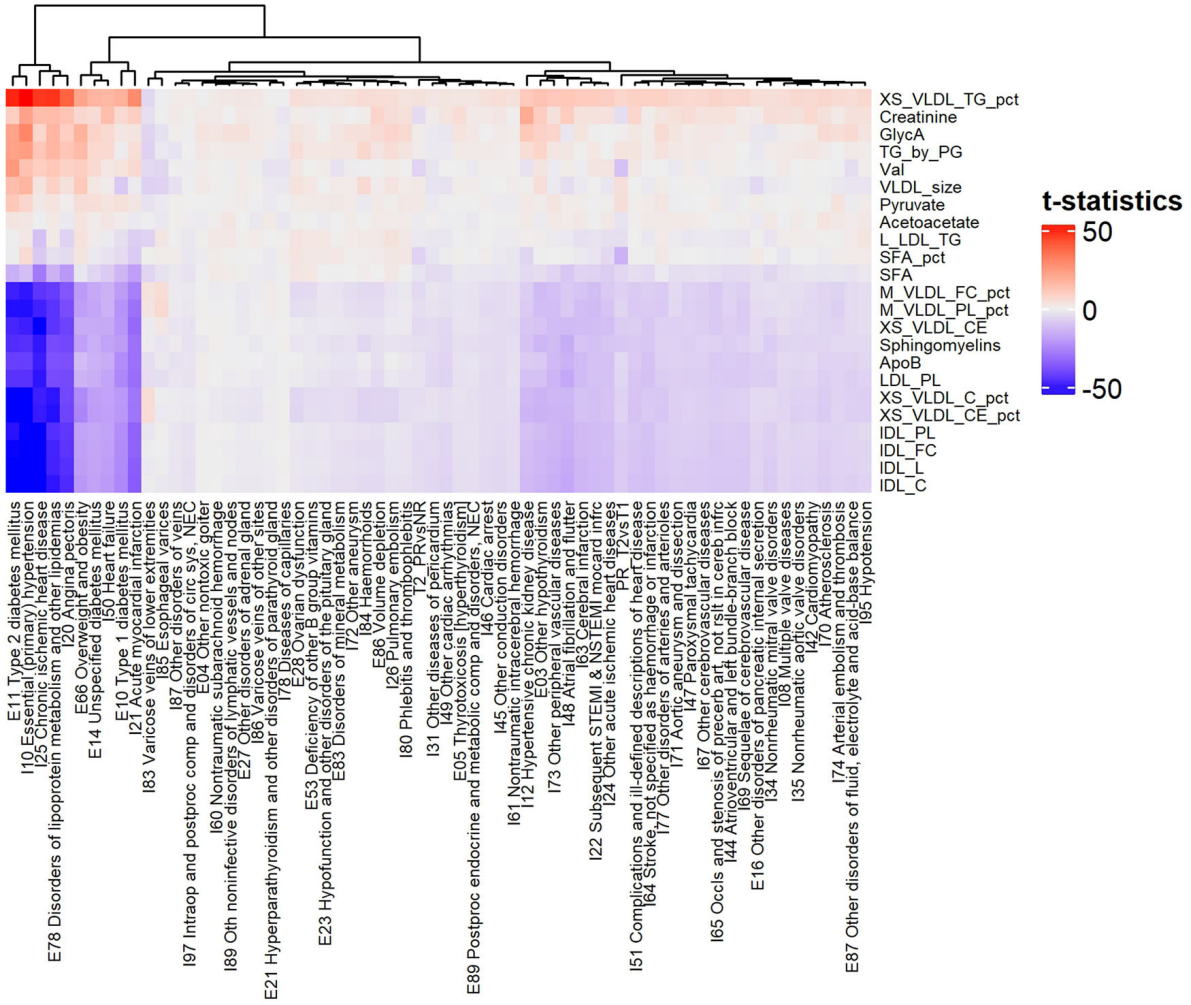
Heatmap of metabolite levels associated with animal product restriction (FastBio) and with cardiometabolic diseases (UK Biobank). Disease codes included ICD10 starting with I- or E-. For each of the 23 metabolite classes, the most significant metabolite associated with animal product restriction was selected. Diseases with the most distinct metabolite profiles correspond to type 2 diabetes, chronic ischemic heart disease, essential hypertension, angina pectoris and disorders of lipoprotein metabolism, which are all strongly associated with lipoproteins. In the dendrogram, comparisons of the PR group between timepoints (PR_T2vsT1) and of dietary groups at T2 (T2_PRvsNR) each form their own distinct branches within their respective clusters. This arrangement highlights specific patterns of associations for PR and T2 that are distinct from those observed with prevalent cardiometabolic diseases in the UK Biobank.

To investigate the impact of animal product restriction-associated metabolite profiles on health, we applied a 14-metabolite score that reflects risk for all-cause mortality, with lower scores associated with a lower mortality risk^26^. Nine of the 14 metabolites examined were detected at altered levels upon dietary restriction (VLDL size, PUFA by MUFA ratio, histidine, leucine, valine, phenylalanine, acetoacetate, XXL_VLDL_L, S_HDL_L). Animal product restriction led to a significant decrease in the mortality score (mean score 0.27 at T1 vs −0.78 at T2, *p* = 9.45e-3), that was not found in the control group (mean score −0.07 at T1 vs 0.58 at T2, *p* = 0.43) (Supplementary Fig. S7). To further address the impact of restriction of animal products, we selected the most significant metabolite from each metabolite class and compared to associations between metabolite levels and cardiometabolic traits (“diseases of the circulatory system” and “endocrine, nutritional and metabolic diseases”) from the UK Biobank^27^. Animal product restriction associations clustered separately from disease associations (Fig. 4) and this was driven chiefly by valine, SFA %, and creatinine, while cardiometabolic outcomes, including acute myocardial infarction and chronic ischemic heart disease, diabetes, obesity, and dyslipidemia strongly associated with lipoprotein profiles (Fig. 4). Accordingly, metabolite profiles associated with an increased risk of these diseases mapped away from restriction-related metabolites profiles (Supplementary Fig. S8).

### Animal product restriction is associated with changes in the levels of multiple plasma proteins

**Fig. 5:**
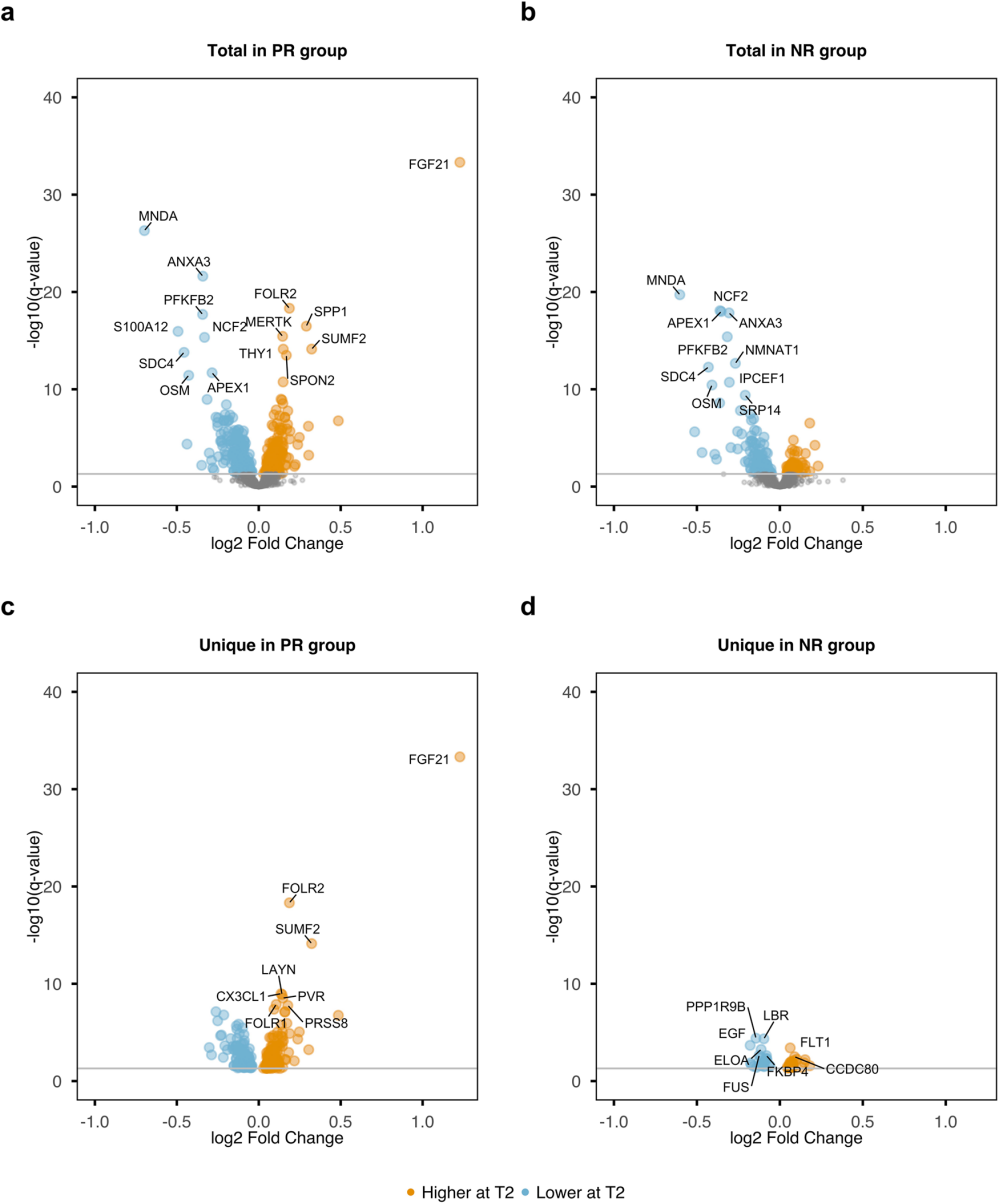
Differentially abundant proteins detected from T1 to T2 for each dietary group. Total differentially abundant proteins detected in the PR group (**a**) and in the NR group (**b**). Unique differentially abundant proteins detected in the PR group (**c**) and in the NR group (**d**). Proteins with increased levels at T2 are shown in yellow whereas proteins with decreased levels are shown in blue. Proteins shown in grey are not significant.

We next sought to investigate how animal product restriction affects plasma protein levels. We quantified levels for 1,472 proteins and following QC (Supplementary Fig. S1, Supplementary Fig. S2), we report results for 1,464 proteins from 793 individuals (199 PR at T1 and 191 PR at T2; 208 NR at T1 and 195 NR at T2). We detected 412 and 195 proteins whose levels changed from T1 to T2 in the PR and NR groups respectively (Fig. 5, Supplementary Data 2). Protein level changes unique to each group were over five-fold higher in PR (268) compared to NR individuals (51), with increased levels of FGF21 being the most prominent change recorded, and unique to the PR group. In addition to PR-unique changes, we found that 144 proteins changed levels in both groups, with the same direction of effect, some of which likely capture seasonal effects. For example, MNDA and ANXA3, proteins associated with neutrophils displayed decreased levels at T2 in both groups. We^28^ and others^29^ have shown that neutrophils, display strong seasonal effects, with their numbers decreasing during spring. Changes unique to the NR group were of lower effect size and significance and may also represent seasonal changes that are suppressed in PR individuals through dietary restriction. For example, the most significantly altered NR-unique protein, PPP1R9B is a substrate for GSK3β, a crucial circadian clock regulator^30^ while PCSK9, a target for LDL-lowering drugs, displays diurnal variation, with levels of daylight duration likely driving seasonal effects^31^.

We also examined differences in protein abundance between dietary groups. Similarly to metabolites quantified, protein profiles at T1 were identical between PR and NR individuals. At T2 however we detected 27 proteins displaying different levels between dietary groups (Supplementary Text S3, Supplementary Fig. S9). Of these, 19 were also detected in the PR group from T1 to T2, adding to the idea that these differences are linked to animal product restriction.

Similarly to the metabolite analysis, we conducted a sensitivity analysis to determine effects of blood pressure on protein abundance. In line with findings on metabolites, we observed that the effect of this correction on our findings was minimal (Supplementary Fig. S10).

We next explored links between metabolites and proteins using unsupervised correlation analysis and detected two correlation modules. The first component displayed correlations with IDL particles and included animal product restriction-associated proteins (FGF21, HAVCR1, ESM1, SPP1, SPON2) thus capturing dietary restriction-associated effects. The second component captured effects not linked to animal product restriction with five proteins (LDLR, NPY, AGRP, CD38, MFGE8) correlating positively with L and XL VLDL particles and with triglycerides (Supplementary Text S4, Supplementary Fig. S11).

### Proteins associated with animal product restriction are druggable targets

Given that interactions between drugs and their targets affect drug action, we sought to determine how many of the proteins whose abundance is altered following animal product restriction (*N* = 264) are targets for known drugs. We found that 62 (23%) proteins are targets for phase 1–4 drugs, and of these 28 (11%) are targets for phase 4, approved drugs (Supplementary Fig. S12). Indications covered by the above drugs include mostly conditions associated with aging such as cancer, cardiovascular, immune system and inflammatory, and visual system diseases (Supplementary Fig. S12, Supplementary Data 2). Notably, over half of the druggable proteins were found to be targets for multiple drugs, with EGFR for example being targeted by 56 phase 1–4 drugs, of which 20 are approved therapeutics for the treatment of various cancer types (Supplementary Fig. S12, Supplementary Fig. S13, Supplementary Data 3).

### Multiple proteins displaying the greatest magnitude of change upon animal product restriction are being evaluated in clinical trials

**Fig. 6:**
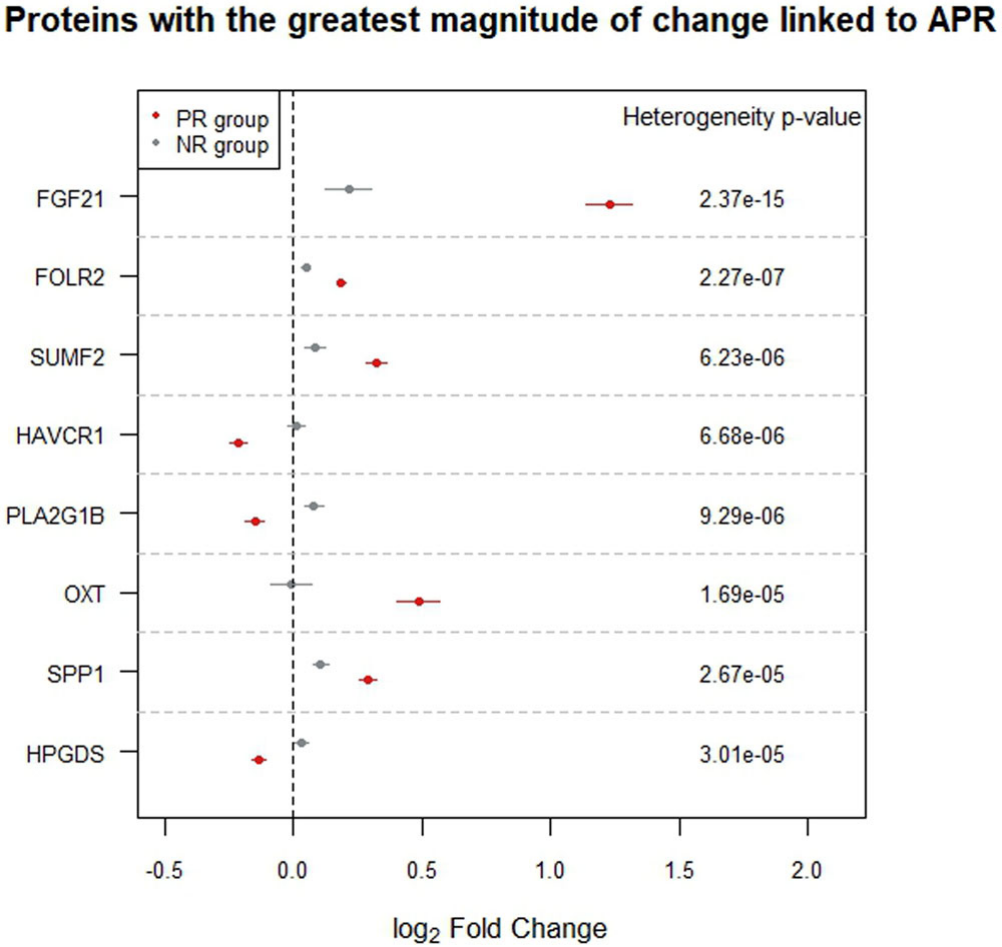
Proteins displaying the greatest magnitude of change linked to animal product restriction. Eight proteins showing the greatest effect size changes from T1 to T2 and between dietary groups were identified through heterogeneity analysis using the Cochran’s statistics (associated p-value indicated in ‘Heterogeneity p-value’). Associations between levels of each protein and timepoints are shown in red for the PR group and in grey for the NR group.

Due to pre-enrichment for specific biological functions of the panel of assayed proteins, we were underpowered to uncover over-represented pathways for proteins detected at altered levels from T1 to T2 (Supplementary Text S5). To focus on proteins most affected by restriction of animal products, we conducted a heterogeneity analysis that aimed to identify proteins for which the effect size of change from T1 to T2 was significantly different between the two dietary groups. Following correction for 1432 independent tests, we highlight eight proteins most affected by animal product restriction: FGF21, FOLR2, SUMF2, HAVCR1, PLA2G1B, OXT, SPP1, and HPGDS (Fig. 6, Table 1). Of these, FGF21, FOLR2 and HAVCR1 were detected at significantly different levels in permanent vegetarian and vegan individuals compared to meat-eaters, in a recent study^7^, with differences being in the same direction as the effects reported here. Using a more permissive 5% FDR correction, we identify 15 additional proteins and underline that over half of these (Table 1) are currently being evaluated in clinical trials for their pharmacological value for age-related outcomes including type 2 diabetes, obesity, cardiovascular disease, cancer, and renal function (Table 1).

**Table 1:** Completed and ongoing clinical trials for proteins showing greatest magnitude of change upon animal product restriction

Protein	No. of clinical trials	Indicative diseases and conditions
**FGF21**	39	gestational and type 2 diabetes, insulin resistance, obesity, liver health
**FOLR2**	0	
**SUMF2**	0	
**HAVCR1**	24	renal function
**PLA2G1B**	54	atherosclerosis, coronary disease, renal function, type 2 diabetes
**OXT**	773	pregnancy and labour, infertility, bleeding, obesity, neurological disorders, substance dependence, pain, social cognition
**SPP1**	25	cancer, infant growth and development
**HPGDS**	1	pulmonary health
**CNDP1**	0	
**PRSS8**	0	
**BAG6**	1	hemoglobin sickle cell disease
**FGF23**	108	renal function, cardiovascular health, metabolic health, X-linked hypophosphatemia
**ASAH2**	0	
**LRIG1**	0	
**PVR**	0	
**THY1**	0	
**FOLR1**	58	fallopian tube, ovarian, breast, cervical and peritoneal cancer
**CTRC**	0	
**DDR1**	2	advanced cancer
**FST**	7	cardiovascular health, muscular dystrophy, infertility, cancer
**FGFR2**	57	gastric and bile duct cancer
**ACE2**	55	COVID-19, cardiovascular health, renal function
**MVK**	5	mevalonate kinase deficiency, Behcet’s disease

### Pro-longevity hormone and potent metabolic regulator FGF21 is the protein most affected by animal product restriction

Our most prominent finding associated with animal product restriction was an increase in levels of liver-derived hormone FGF21. Although FGF21 is well-studied in model organisms, its biology in humans remains poorly understood^32^. In humans and mice, FGF21 has key roles in regulation of energy homeostasis, lipid and glucose metabolism, and insulin sensitivity^2,33,34^. Moreover in mice, prolonged overexpression of this hormone has been shown to extend lifespan^35^. FGF21 is induced by dietary protein restriction^36^ and by restriction of specific amino acids^2^ and mediates its effects directly on adipose tissue and through signalling to the brain to regulate macronutrient preference^37^. Work in rodents and non-human primates has demonstrated substantial pharmacological benefits of FGF21 on a range of metabolic complications linked to obesity, including a reduction in fat mass and alleviation of hyperglycaemia, insulin resistance, dyslipidaemia, cardiovascular disorders and non-alcoholic steatohepatitis (NASH)^32^. Clinical trials of several FGF21 analogues and mimetics (Supplementary Data 4) have led to considerable improvements in conditions including dyslipidaemia, hepatic fat fractions and serum markers of liver fibrosis in NASH patients, but the primary end points of glycemic control have not been met, suggesting divergence of FGF21 biology in humans^32^.

FGF21 signalling in humans therefore remains to be elucidated and its complexity may also be exacerbated by the existence of FGF21 inactivation enzymes^33^ such as FAP. We found that upon animal product restriction FAP levels decreased, potentially resulting in extension of the short half-life of FGF21 (0.5–1.5 h). To address the complexity of FGF21 signalling more broadly, we conducted differential abundance analysis on the 411 proteins associated with animal product restriction (excluding FGF21), and adjusted additionally on FGF21 levels. Results did not change for the NR group, but in the PR group, 55 proteins were no longer significant, suggesting association of their abundance with FGF21 (Supplementary Data 5). Evidence for links to FGF21 exists for some of these proteins (e.g. FGF23, EGFR and CCL2) (Supplementary Fig. S14), but for others, their connection to FGF21 remains unknown, rendering them good candidates to investigate for their role in FGF21 signalling in humans.

### Animal product restriction is associated with increased abundance of proteins with key metabolic functions

In addition to FGF21, increased levels upon restriction were recorded for FOLR2, SUMF2 and OXT. FOLR2 imports folate to the interior of cells and together with vitamin B12, a micronutrient found exclusively in animal products, is an essential compound of the folate and methionine cycles^38^. Deficiency of vitamin B12, typical in vegan individuals^6^, can mimic the effects of folate deficiency. Given that under conditions of folate deficiency, folate receptors are upregulated^39^, we suggest that restriction-associated increased FOLR2 levels may be in part due to decreased vitamin B12 intake. SUMF2 is an inhibitor of the enhancing effects of SUMF1 on sulfatases^40^ and causal effects on decreased levels of HDL, LDL and total cholesterol have been found^41^. Restriction of animal products therefore has likely inhibitory effects on sulfatase activity through increased levels of SUMF2, and may contribute to effects on the above lipids. OXT is a hypothalamic hormone known for its roles in parturition and social bonding, but also acts as a sensor of nutrient status^42^. In humans, decreased circulating OXT levels have been recorded in individuals with obesity or diabetes, and OXT administration is currently being explored as a treatment for obesity-related comorbidities^43^ (Supplementary Data 4). Similarly to FGF21^37^, OXT shapes macronutrient preference and affects weight control^42^.

SPP1 (OPN) was detected at increased levels in both dietary groups from T1 to T2, but the magnitude of change was greater for PR individuals. SPP1 has diverse functions including bone calcification, cell survival, proliferation and migration, and immune function^44^ and is in clinical trials for indications linked to cancer (Table 1). SPP1 expression is induced by calcitriol, the hormonal metabolite of vitamin D, that is produced under low levels of calcium^44,45^. Given the impact of sunlight on vitamin D, increased levels of SPP1 observed for both dietary groups from T1 to T2, may be linked to seasonal effects. Furthermore, individuals practicing restriction of animal products typically have lower calcium intake^11^, as do vegans, who display consistently lower levels of vitamin D^6^. The greater magnitude of change in SPP1 levels in the PR group may therefore be due to a combination of seasonal and dietary restriction-related effects.

### Animal product restriction is associated with decreased abundance of proteins with immunometabolic functions

Decreased levels upon restriction were detected for HAVCR1, PLA2G1B and HPGDS. HAVCR1 is a transmembrane receptor with roles in regulation of immune cell activity and renal regeneration^46^. It is abnormally expressed in a range of tumours, upregulated during renal injury^46^, and is currently in multiple clinical trials mostly for indications linked to renal function (Supplementary Data 4). In rat primary renal tubular epithelial cells, administration of the saturated fatty acid (SFA) palmitate results in upregulation of HAVCR1^47^. Decreased HAVCR1 levels are therefore consistent with the prominent reduction of SFA% detected upon restriction of animal products, reflecting the lower intake of saturated fats^25^. HAVCR1, which is expressed predominantly on the surface of T helper 2 (Th2) cells, has been shown to increase the immune response by promoting activation and proliferation of T cells and cytokine secretion^48–50^. A decrease in HAVCR1 levels therefore likely has a tempering effect on inflammation, with inhibition of HAVCR-mediated signalling constituting a new type of anti-tumour therapy^51^. PLA2G1B is a secreted phospholipase A2 produced by pancreatic acinar cells^52^, and is currently being investigated in clinical trials for a range of cardiometabolic conditions (Supplementary Data 4). *Pla2g1b* knock-out mice display reduced phospholipid digestion and concurrent attenuation of diet-induced obesity, insulin resistance, and atherosclerosis. Conversely, pancreatic acinar cell-specific overexpression of Pla2g1b has been linked to increased phospholipid digestion and to exacerbation of diet-induced obesity and insulin resistance^52^. Notably, members of the phospholipase A2 family have been linked to healthspan and longevity in humans in a study showing that PLA2G7 downregulation in adipose tissue mediated the beneficial effects on health of prolonged caloric restriction^16^. Finally, HPGDS is an enzyme that catalyses the conversion of PGH2 to PGD2. PGD2 is a prostaglandin that acts as an early-phase mediator of inflammation^53^ and augments disease activity, for example in asthma^54^. Decreased levels may therefore contribute to a tempering effect on inflammation and the role of HPGDS in pulmonary health is currently being evaluated in clinical trials (Supplementary Data 4). Both PLA2G1B and HPGDS have key roles in the production of inflammatory prostaglandins through the arachidonic acid (AA) pathway^55–58^ and their decreased levels likely result in a decrease of prostaglandin production. Pharmacological inhibition of the cyclooxygenase branch of the AA pathway and subsequent suppression of prostaglandin production is a promising therapeutic approach^59^ with drugs for multiple diseases, including rheumatoid arthritis (RA) having already been approved^60,61^. Furthermore, vegan diets, which are known to ameliorate RA symptoms, also likely mediate their effects partially through reduction of prostaglandin production, among other changes^62,63^. In line with the above, we have shown that animal product restriction was linked to a ~ 73% decrease in normal range CRP concentration^28^. This decrease may be partially driven by a reduction in prostaglandins, given their role in promoting the release of pro-inflammatory cytokines and subsequent CRP production^64,65^.

### Proteins affected by restriction of animal products have potentially causal effects on cardiometabolic diseases

**Fig. 7:**
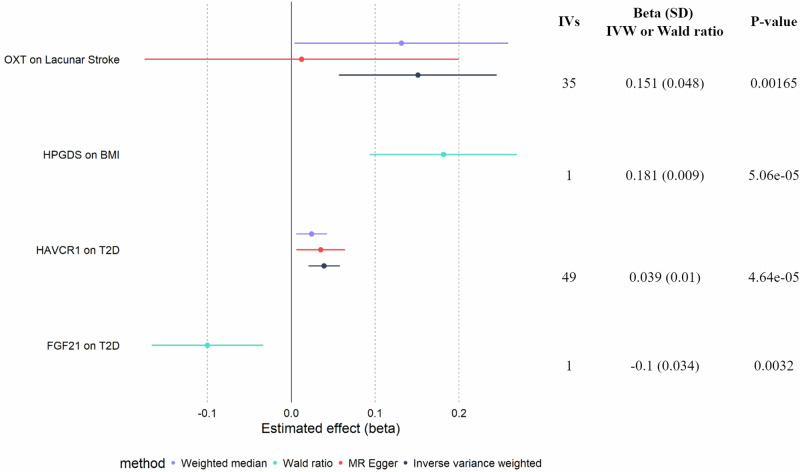
Forest plot representing the effect sizes of two-sample MR for significant proteins-cardiometabolic traits. The IVW method along with sensitivity analyses are shown for proteins with more than one IV, and the Wald ratio estimates are shown for proteins with only one IV.

Given the key roles of the eight proteins above, we sought to investigate causal effects of their genetically regulated levels on cardiometabolic traits using Mendelian randomization (MR). We focused on traits and diseases from the UK Biobank with associated metabolite profiles that mapped the furthest away from metabolite profiles of PR individuals (Supplementary Fig. S8), namely type 2 diabetes, body mass index (BMI, proxying obesity), coronary artery disease, lacunar stroke (proxying atherosclerosis) and essential hypertension. At the Bonferroni corrected significance threshold, we identified a protective effect of FGF21 and HACVR1 on T2D risk and a causal effect of HPGDS on decreased BMI (Fig. 7, Supplementary Data 6). We also found that increased OXT levels drive an increased risk for lacunar stroke, which is in the opposite direction of the suggested protective effects of OXT against cardiovascular risk in the literature^66^. However, vegetarian individuals from the EPIC-Oxford study display an increased risk of stroke compared to meat-eating individuals^6^.

## Discussion

In the present study, we performed plasma metabolomic and proteomic profiling for the first time on a group of individuals who follow a unique dietary pattern, and have revealed extensive metabolic reprogramming associated with abstinence from meat, fish, dairy products and eggs. This dietary behaviour comprises a type of intervention practiced voluntarily for religious reasons, and results in overall lower intake of protein, but not of energy^10,11^.

Animal product restriction-associated changes in metabolite levels resulted in profiles compatible with a positive impact on health. Metabolite profiles linked to restriction clustered separately from metabolite profiles associated to cardiometabolic disease, with lipids, amino acids and fatty acid % particularly driving these differences. In addition to reductions in levels of multiple lipid classes, we found reductions in mTORC1 agonists valine and leucine. mTORC1 integrates nutrient and hormonal cues to regulate cellular processes linked to growth and proliferation, and ultimately healthspan and longevity^2,3^. Methionine restriction also downregulates mTORC1 activity, driving increased levels of FGF21 and improved metabolic profiles^2,24,36^. Given that animal products are rich in methionine, and also that vegetarian and vegan individuals typically displaying lower plasma levels of this amino acid^7,67,68^, we suggest that the observed increase in FGF21 levels may also be driven by reduced intake of methionine. Overall, changes in protein levels associated with dietary restriction were in a direction suggesting mostly favourable effects on health, further confirmed through MR for FGF21, HAVCR1 and HPGDS.

In addition to positive effects on health we also find effects that are potentially negative. Increased levels of FGF21, but also of restriction-associated changes in levels of OXT, SPP1, FGF23, PTH1R and PTHrP, may exert detrimental effects on bone homeostasis^32,42,69^. We have also revealed an effect of OXT on increased risk for lacunar stroke. Increased risk of stroke in vegetarians has been reported previously^6^, but the mechanisms underlying this observation are not clear. Proposed explanations include low levels of LDL cholesterol, high levels of homocysteine due to low vitamin B12, and low intake of animal protein^6^, but further investigation is required to understand how risk is determined. Furthermore, due to their role in regulating dietary preference, impaired signalling through FGF21 and OXT underlies dietary choices that exacerbate obesity and linked diseases^70^. Understanding the signalling pathways driven by these hormones will aid in harnessing the mechanisms that govern feeding regulation and will contribute to the development of FGF21 and OXT-based pharmacotherapies that promote healthy diet choice.

Our work has highlighted high-value candidates to evaluate for their potential therapeutic effects and that can inform the development of drugs mimicking molecular responses that promote healthy aging. In addition to the potential of restriction-associated proteins as pharmaceuticals, we have also revealed changes in the abundance of potentially druggable proteins suggesting that dietary interventions can impact the kinetics of drug-target binding^71^ and may be used to fine-tune the action of drugs.

Although the molecular changes we have uncovered are extensive, they appear to be transient. This is true for metabolites and proteins quantified, where profiles between dietary groups were identical when both groups were on an omnivorous diet. Our study design has also enabled us to uncover changes in protein abundance that are likely seasonal (e.g. neutrophil-associated proteins MNDA and ANXA3) and that remain understudied in humans despite known seasonal trends of diseases including cardiovascular disease, allergies, autoimmune conditions, and psychiatric disorders^72^.

This work is limited by the absence of dietary intake data for study participants. However, given the very specific guidelines outlined by the Greek Orthodox Church, the dietary pattern under study is highly consistent between different groups of practicing individuals. Indeed, for overlapping measurements, our findings are in line with those from two studies covering the same restriction period (Lent)^10,11^. Secondly, profiling was not performed immediately prior to initiation of animal product restriction. However, given that the dietary patterns preceding both T1 and T2 are very similar^28^, we expect that measurements prior to initiation of dietary restriction at T2 would have been very similar to measurements at T1. Finally, molecular levels assessed were in plasma samples, thus representing the biology of multiple tissues and not enabling us to ascertain tissue-specific mechanisms.

In summary, our results suggest that animal product restriction results in rapid and extensive molecular reprogramming with mostly positive, but some likely detrimental effects on health. Further work is required to better understand how underlying molecular mechanisms including changes in gene transcription, in immune cell type composition and in the gut microbiome, affect mechanisms linked to cardiometabolic health, aging and risk for disease. Furthermore, longitudinal studies are required to map long-term effects on health and to evaluate whether simple dietary supplementation (e.g. with vitamin B12) can minimise negative consequences. Finally, detection of sex- and age-related effects of animal products restriction will contribute towards tailored approaches in the context of personalized medicine and nutrition.

## Methods

### FastBio population sample

The FastBio study was advertised during Autumn 2017 to the local communities of Thessaloniki, Greece through various means, including the press, radio, social media, and word of mouth. Candidate participants expressed their interest via telephone, email or via the project’s website (www.fastbio.gr). Following screening of over 1000 candidates from the greater area of Thessaloniki through two interviews, 411 apparently healthy unrelated individuals, who met FastBio selection criteria (Supplementary Text S1) were included in the study. Participants belonged to one of two groups, specified by their diet: 200 individuals who followed a temporally structured dietary pattern of animal product restriction (periodically restricted, PR group) and 211 continuously omnivorous individuals who follow the diet of the general population and were not under any kind of special diet (non-restricted group, NR group).

PR individuals constitute a unique study group given their consistent adherence to a specific dietary pattern that involves alternating between omnivory and abstinence from meat, fish, dairy products and eggs (but not shellfish and molluscs) for 180–200 days annually (for detailed restriction pattern see Supplementary Text S2, Fig. 1b). The PR diet is practiced for religious reasons as specified by the Greek Orthodox Church and is typically part of the culture of the family PR individuals are born into. It is initiated during childhood and is temporally structured with a specific periodicity involving four extended periods of restriction throughout the year, as well as restriction on Wednesdays and Fridays of each week. During periods of restriction, PR individuals also typically consume less alcohol. When not practicing restriction, PR individuals follow an omnivorous type of diet similar to the general population. Only PR individuals who had followed this dietary pattern for at least ten years were included in the study.

The FastBio population sample is described in Loizidou et al. ^28^ briefly, compared to NR individuals, PR were on average older (PR: 51.5 ± 13.5, NR: 45.0 ± 13.1), had higher BMI (PR: 28.4 ± 4.6, NR: 26.2 ± 4.4) and were less likely to smoke (smokers PR: 6.5%, NR: 33.2%). Both groups had similar systolic (SBP) and diastolic blood pressure (DBP) (SBP PR: 127 ± 19.0, NR: 121 ± 19.7; DBP PR: 80 ± 10.8, NR: 78 ± 11.8). and consisted of slightly more female participants (PR: 54%, NR: 55%). Participants were from families living above the line of poverty, were mostly married (PR: 73.5%, NR: 65.9%), had tertiary education (PR: 67%, NR: 75.4%), and originated mostly from Northern Greece (PR: 68.5%, NR: 65.4%) (Supplementary Data 1). Previous work from our group and others on the Greek population has shown that Greek individuals display similar patterns of genetic diversity to individuals from Southern European, with Greek individuals mapping close to individuals of Italian and Spanish ancestry^73–76^. Levels of physical activity were similar between groups and timepoints and the vast majority of participants were apparently healthy (according to self-reports) although a minority had underlying chronic conditions, including diabetes, arterial hypertension and hypothyroidism, for which treatment was being received. Very few participants were taking dietary supplements^28^.

### Collection of data, of biological material and measurement of traits

All FastBio participants were invited to two scheduled appointments at the Interbalkan Hospital of Thessaloniki. The first appointment took place in October-November 2017 (Timepoint 1 (T1)) and covered a period during which PR individuals had been on an omnivorous diet for 8-9 weeks (excluding Wednesdays and Fridays). The second appointment (Timepoint 2 (T2)) took place in March 2018 and was during Lent, covering a period where PR individuals had abstained from meat, fish, dairy products and eggs for at least three and a maximum of four weeks. A recall rate of 95% was achieved at T2, with 192 (out of 200) PR participants and 198 (out of 211) NR participants attending both appointments. For both timepoints, all appointments were scheduled between 7:30-9:30 am to minimize circadian effects, and were completed during a two-week window to minimize effects of seasonality for the specific sampling window. All participants gave their written informed consent and the study was approved by the local ethics committee (BSRC Alexander Fleming Bioethics Committee Approval 17/02/2017). For each participant, following overnight fasting, blood was drawn into EDTA-coated tubes and centrifuged to obtain plasma within 10–15 min of collection. Plasma was stored at -80°C. Aliquots were taken forward to generate metabolomics and proteomics data as described below. Clinical chemistry measurements were performed as described in ref. ^28^ .

### Metabolomic profiling

For each sample, a plasma aliquot of 100 μL was used for metabolomics analysis. Profiling of 249 metabolites was through high-throughput NMR spectroscopy (Nightingale Health Plc, biomarker quantification library 2020), a technology that enables precise absolute quantifications for most of the measured metabolites and that has been widely used in studies including the UK Biobank^27^. The Nightingale panel is mostly composed of lipids and includes 168 absolute metabolite levels and 81 derived measures (ratios and percentages). Among the 801 samples, two could not be quantified, one due to a lack of material and one due to a technical error. Raw data corresponding to absolute concentrations or ratios of metabolites for the 799 remaining individuals were used for further analysis. To compare Nightingale NMR biomarker measurements with standard clinical chemistry measurements, we performed correlation analysis for total cholesterol, total triglycerides, HDL and LDL cholesterol for which both NMR and clinical chemistry measurements were available^28^. We report high congruence between NMR and clinical chemistry measurements (R > 0.86, *P* < 2.2e-16) (Supplementary Fig S15).

### Proteomic profiling

For each sample, a plasma aliquot of 40 µL was used for proteomics analysis. Plasma protein levels were measured on the Olink Explore 1536 platform (Olink Proteomics AB, Uppsala, Sweden) using the Neurology, Oncology, Cardiometabolic and Inflammation 384-plex panels (panel lot number B04414, B04412, B04413, B04411, respectively). Olink Explore is a protein biomarker platform that utilizes Proximity Extension Assay (PEA) technology with next-generation sequencing readout. Data generated from Olink are pre-processed and quality controlled using Olink data analysis software. Protein levels are reported in normalized protein expression values (NPX), which is a relative quantification unit in log_2_ scale with 1 NPX increase corresponding to doubled protein concentration.

### Metabolite and protein quality control

All quality control (QC) steps are summarized in Supplementary Fig. S1. QC procedures and statistical analyses were performed in R (version 4.1.3). Three individuals were removed from the analysis because they were first degree relatives with other participants. The success rate of metabolites and of samples were used to filter the data. The success rate was defined as the proportion of samples for which the metabolites had non-missing values. A threshold of 85% was applied for metabolites, resulting in the selection of 248 metabolite assays, and a threshold of 75% was applied for samples, resulting in the selection of 797 samples. The thresholds were based on the distribution of the QC metrics in the sample. The only metabolite removed was 3-Hydroxybutyrate (success rate 37%). Values below limit of detection (LOD) were kept. Metabolite levels were log_2_-transformed prior to statistical analysis. We used a similar approach for proteins measured by the Olink Assay with the same thresholds. This resulted in the selection of 1464 protein assays and 793 samples. For quality assurance requirements, the panel includes three proteins that are quantified four times each (i.e. some proteins contain multiple IDs), the 1464 protein assays correspond to 1455 actual proteins. For simplicity, the term ‘proteins’ refers to the Olink assays. From the remaining data, all the values that failed either Olink Assay QC or Olink Sample QC were set as missing, while values below LOD were kept in the downstream analyses. NPX values being already in the log_2_ scale, no further transformation of the data was applied prior to statistical analysis.

Principal Component Analysis (PCA) was used to check for sample outliers. Missing data were imputed by the mean prior to the PCA. The PCA on metabolite levels revealed 14 outliers (Supplementary Fig. S2a) as samples with more than three standard deviations from the mean in the two first components (indicated by the red dashed lines). These samples were excluded from further analyses. On the PCA based on protein levels, a homogeneous group with no extreme outliers was observed (Supplementary Fig. S2b). To maximize sample sizes and statistical power, no individual was removed on the basis of this PCA. The final dataset consisted of 777 and 793 samples for the metabolomics and proteomics statistical analyses respectively.

### Statistical analysis

Differential abundance analysis for metabolite and protein levels was performed using the limma package (version 3.50.3)^77^. We used limma to record: A) changes in measured traits for each dietary group between timepoints (positive effects correspond to an increase at T2 compared to T1), and B) differences of measured traits between dietary groups for each timepoint (positive effects correspond to higher levels in PR compared to NR). To take into account the within-individual comparisons in the paired analyses, a linear mixed model was used where dietary groups and timepoints were treated as fixed effects and subject IDs were treated as random effects. PCA on 1464 quantified proteins and on 249 quantified metabolites revealed effects for sex, age squared and BMI (Supplementary Fig. S16 and Supplementary Fig. S17). All analyses were adjusted on sex, age squared, BMI, smoking, and also on medication use (including medication for hypertension, thyroid, diabetes, osteoporosis and antiplatelets). Participants were defined as smokers and non-smokers (with past smokers and e-cigarette users also considered as non-smokers) based on NHS guidelines (https://www.nhs.uk/live-well/quit-smoking/using-e-cigarettes-to-stop-smoking/). Each medication variable was binary reporting whether individuals were taking this medication or not. It is to note that few participants were on medications (ranging from 7 individuals for antiplatelets medication, to 53 for hypertension medication out of the 411 samples at T1). For the proteomics analysis, the mean value of protein levels for each sample was included as an additional covariate. Limma was run separately on metabolite and protein levels and p-values were adjusted for multiple testing using the false discovery rate (FDR). Metabolites and proteins with an adjusted p-value lower than 5% were considered as differentially abundant. To explore whether blood pressure has an impact on our findings, we conducted a sensitivity analysis adjusting additionally for systolic and diastolic blood pressure (SBP and DBP respectively).

### Mortality score

To assess the effect of metabolite profiles on health, we used a mortality score developed by Deelen et al. ^26^ combining levels of 14 selected metabolites from the Nightingale panel (XXL-VLDL-L, S-HDL-L, VLDL size, PUFA by FA ratio, glycine, lactate, histidine, isoleucine, leucine, valine, phenylalanine, acetoacetate, albumin and glycoprotein acetyls). Lower values of the score have been shown to be associated with a decreasing hazard ratio of overall mortality in a cross-sectional study, making it a surrogate for overall health of individuals. Following log_2_-tranformation and scaling, the metabolite score was calculated for FastBio participants at both timepoints in both dietary groups. The scores were then regressed on the same covariates as in the differential expression analysis (age squared, sex, BMI, smoking, hypertension medication, diabetes medication, osteoporosis medication, cholesterol medication and antiplatelets medication) and compared between the groups using linear models.

### Comparisons of animal product restriction-associated profiles to UK Biobank metabolite associations with cardiometabolic diseases

To investigate the potential impact of metabolite changes linked to dietary restriction on cardiometabolic health, we compared the pattern of metabolite associations observed in FastBio to the ones described by Julkunen et al. ^27^ with multiple cardiometabolic traits from the UK Biobank cohort (summary statistics downloaded from https://biomarker-atlas.nightingale.cloud/). We focused on ICD10 codes starting with ‘I-‘ and ‘E-‘, which correspond respectively to “diseases of the circulatory system” and to “endocrine, nutritional and metabolic diseases”, totalling 62 diseases. The t-statistics were computed from the estimates and standard errors, available in the downloaded file. We did not include the t-statistics of the comparisons within the NR group (T2 vs T1) and at T1 (PR vs NR) as we detected only one and zero significant metabolites respectively. To explore whether specific metabolite classes drive differences between animal product restriction-related profiles and cardiometabolic disease-related profiles, we selected the most significant metabolite from each of the 23 classes (as depicted in Fig. 2) and clustered association patterns. We used R package ComplexHeatmap. ^78^ to represent direction of associations. We then performed PCA using the prcomp function in R, on the full metabolite profiles of associations driven by dietary restriction (PR group T2 vs T2; T2 timepoint PR vs NR) and with cardiometabolic disease.

### Identifying proteins linked to FGF21 signalling

We investigated potential effects of FGF21 on proteins that were found to be differentially abundant between timepoints as a sensitivity analysis. For this, we repeated the differential protein expression analysis as described above, but restricted to proteins designated as differentially abundant (411, excluding FGF21 in the PR group; 195 in the NR group) in the primary analysis, and adjusted additionally for FGF21 levels. If a previously identified protein was no longer significant (FDR > 0.05) after adjusting for FGF21 levels, we hypothesized that the two signals were not independent. STRING DB (v 12.0) was queried to visualize interactions between FGF21 and proteins that did not retain their significance.

### Animal product restriction-associated proteins as drug targets

We queried Open Targets^79^ (March 8th 2024) to investigate whether differentially abundant proteins unique to the PR group are drug targets. For each differentially abundant protein that was a target for phase 1-4 drugs, we retrieved information on phase and status of the clinical trial, the approval status of the associated drugs, and their indications. Clinical trials with “Terminated”, “Withdrawn” or “Unknown” status were excluded. We focused on the analysis of phase 4 and approved drug-differentially abundant protein pairs and grouped their indications into broader Experimental Factor Ontology (EFO) disease categories to further explore their clinical translatability. Diseases categorized under the EFO terms ‘other disease’ or ‘other trait’ were further grouped, where possible, using the ancestral diseases as retrieved from Ontology Lookup Service (OLS) using the ‘rols’ R package^80^.

### Protein level heterogeneity analysis

To uncover proteins displaying the greatest magnitude of animal product restriction-associated change, we performed a heterogeneity analysis on the betas obtained from the limma association models. For this purpose, the Cochran’s statistics was used as follows:

$${Q}_{{{stat}}_{T2{vsT}1}}=\frac{{\log {FC}}_{{PR}}* {w}_{{PR}}+{\log {FC}}_{{NR}}* {w}_{{NR}}}{{w}_{{PR}}+{w}_{{NR}}}$$

Where ${\log {FC}}_{{PR}}$ corresponds to the log-fold change of the differential analysis between timepoints in the PR group (and in the NR group respectively), and ${w}_{{PR}}$ corresponds to the weight given to the analysis in the PR group which depends on the standard error ${{se}}_{{PR}}$ as:

$${w}_{{PR}}=\frac{1}{{{se}}_{{PR}}^{2}}$$

${w}_{{NR}}$ is defined similarly for the NR group.

This statistic follows a chi-square distribution with one degree of freedom. To assess the statistical significance of the test, we used a Bonferroni-corrected significance threshold. We computed the effective number of proteins based on their correlation pattern using the procedure described by Li and Ji^81^, giving a total of 1,432 independent tests. We then divided the classical 5% threshold by this value, resulting in a significance threshold of 3.5e-5.

### Animal product restriction-associated proteins in clinical trials

We queried https://clinicaltrials.gov/ (Oct 30th 2023) for the top 23 most heterogenous differentially abundant proteins (FDR < 5% in the heterogeneity analysis) inputting the protein name in the intervention/treatment field and reporting all clinical trials on record (phases 1–4).

### Causal effects of proteins associated with animal product restriction on cardiometabolic traits

To investigate the causal effect of the eight proteins Bonferroni-significant in the heterogeneity analysis on age-related traits, we performed two-sample Mendelian randomization (MR). We focused on five complex traits: T2D, body mass index (BMI), coronary artery disease (CAD), lacunar stroke and essential hypertension. We selected the instrumental variables (IVs) for the eight proteins by using the protein quantitative trait locus (pQTLs) described in the UK Biobank^82^, available in https://www.synapse.org/#!Synapse:syn51364943/files/. To match the ancestry of the FastBio cohort, we used the summary statistics from the European population. We focused on cis-pQTLs by defining windows at +/- 500 kb from the genomic positions of the corresponding genes, obtained through GeneCards (Supplementary Data 6). We then performed clumping by using the 1000Genomes European reference panel, a r2 threshold of 0.001 in 10 Mb windows, and a p-value threshold of 1e-5. The F-statistic for the remaining variants was computed as ${\beta }^{2}/{{se}}^{2}$, and only variants with an F-statistic greater than 10 were considered as IVs. The summary statistics for the five complex traits were downloaded from publicly available databases, by minimizing sample overlap with the UK Biobank (Supplementary Data 6). We then performed two sample MR by using the package TwoSampleMR^83^. We considered the inverse variance weighted (IVW) method, or the Wald ratio if only one IV was present, to assess statistical significance at a threshold of 1.61e-3, corrected for the 31 tests performed. To assess robustness of the results against the MR assumptions, we verified the direction of effect obtained using the weighted median and MR-Egger methods. Reported significant signals correspond to signals with a significant *p*-value and a concordant direction of effect with the sensitivity analyses. If heterogeneity was present (nominally significant *p*-value), we further applied MR-Presso^84^ and considered the corresponding p-value after removing outliers^85^.

## Supplementary information

Supplementary information

Supplementary Data1

Supplementary Data2

Supplementary Data3

Supplementary Data4

Supplementary Data5

Supplementary Data6

## Data Availability

Metabolomics and proteomics data have been deposited in Zenodo https://zenodo.org/uploads/14687361.
