# Supplementary material for: Periodic dietary restriction of animal products induces metabolic reprogramming in humans with effects on cardiometabolic health
Source: NPJ Metab Health Dis. 2025 Apr 9;3:14. doi: 10.1038/s44324-025-00057-2 (PMC11981922; doi:10.1038/s44324-025-00057-2)
Supplement: Supplementary file 1 — Supplementary information [file 44324_2025_57_MOESM1_ESM.docx]

**Supplementary Information**

**Supplementary Text S1. FastBio study inclusion and exclusion criteria**

Inclusion criteria:

- Healthy female and male subjects 18 – 75 years of age at the time of enrolment. One participant turned 76 between time of enrolment and first sampling timepoint (T1).
- Able to provide signed and dated informed consent. Willing to provide blood samples.
- Individuals who had practiced periodic animal product restriction for at least ten years (for the PR group).
- Individuals who had not practiced any kind of specific diet including veganism, vegetarianism, caloric restriction, intermittent fasting (for the NR group).

Exclusion criteria:

- Use of antibiotic, antifungal, antiviral or antiparasitic drugs six months prior to T1.
- Acute disease, defined as the presence of a moderate or severe illness with or without fever, at sampling timepoints.
- Alcohol or drug abuse two years prior to T1.
- Participants who are normally periodically abstaining from animal products, but had to alter their diet for a specific reason (e.g. pregnancy).

**Supplementary Text S2. Dietary pattern of periodic animal product restriction specified by the Greek Orthodox Church**

Individuals following the dietary regimen of the Greek Orthodox Church practice restriction of animal products through abstinence from meat, fish, dairy products and eggs for 180-200 days annually. Consumption of shellfish and molluscs is permitted on all days of restriction. Restriction is practiced on Wednesdays and Fridays throughout the year (excluding the week immediately after Christmas, Easter and the Pentecost) and over four extended periods annually as follows (**Fig. 1b**):

- 40 days before Christmas (abstinence from meat, dairy products and eggs, consumption of fish is permitted except on Wednesdays and Fridays).
- 48 days before Easter (Lent) (abstinence from meat, fish, dairy products and eggs, consumption of fish is permitted only on March 25^th^ and on Palm Sunday).
- 0-30 days in June (abstinence from meat, dairy products and eggs, consumption of fish is permitted).
- 15 days before August 15th (Assumption) (abstinence from meat, fish, dairy products and eggs, consumption of fish permitted only on August 6^th^).

FastBio participants were profiled during a period of omnivory (T1, prior to Christmas) and during a period of animal product restriction (T2, during Lent). Typically, during periods of omnivory, protein accounts for ~15% of total energy intake ^1, 2^. During Lent however when practicing individuals abstain from meat, fish, dairy products and eggs, protein accounts for 8.7-10.7% of total energy intake. Protein intake during this period is largely through consumption of plant-derived protein sources such as legumes. Therefore, during this restriction period, individuals are consuming lower levels of protein and most protein is from plant-based products.

**Supplementary Text S3. Comparison of metabolite and protein profiles between dietary groups at T1 and T2**

At T1, when both groups were on an omnivorous diet, no differences in metabolite levels were detected **(Supplementary Fig. S4, Supplementary Fig. S5, Supplementary Data 2**). At T2, following dietary restriction for the PR group, we report 86 metabolites found at significantly different abundance between dietary groups. Of these, 83 (97%) were also detected in the PR group from T1 to T2. Furthermore, at T1 we find a similar mortality score between PR and NR groups (mean score 0.27 in PR vs -0.07 in NR, p=0.538 (**Supplementary Fig.** **S7**), but at T2 we report significantly lower values for PR individuals (mean score --0.78 in PR vs 0.58 in NR, p=0.02). When comparing the association patterns from T2 with complex diseases, the trends we found were similar to the association profiles comparing the two timepoints in PR individuals (**Supplementary Fig. S4, Supplementary Fig, S5**). Similarly, we examined differences in protein levels between dietary groups and found that 0 and 27 proteins existed at significantly different levels at T1 and T2 respectively (**Supplementary Fig. S9**). Associations at T2 likely reflect acute effects of animal product restriction, with FGF21 being the most significant finding. Of the 27 proteins that were unique to T2, 19 (70%) were also detected as uniquely associated in PR individuals from T1 to T2 supporting the idea of effects linked to animal product restriction.

**Supplementary Text S4. Integration of metabolite and protein datasets**

To explore links between metabolites and proteins we performed unsupervised correlation analysis using the sPLS (Sparse Partial Least Squares) method of the mixOmics package ^3, 4^. This method identifies linear combinations of the variables from each omics dataset and reduces the dimensionality, while also performing variable selection through LASSO penalization. To enhance power, we analysed all participants at both timepoints, accounting for repeated measures and used variable selection to focus on a subset of optimally selected key predictor metabolites and proteins. For the selection of the number of variables per component per dataset, 10-fold cross-validation was repeated five times and the outcome that maximized the correlation between the predicted and the actual components was chosen. Optimal selection involved two components with five variables each. The first component captured animal product restriction-associated effects, with five PR-associated differentially abundant proteins (FGF21, HAVCR1, ESM1, SPP1, SPON2) displaying correlations with IDL particles (**Supplementary Fig. S11**). Although we do not have evidence for causality, we suggest that IDL particles which were affected by dietary restriction, may comprise a promising metabolite type to investigate further for effects on health. The second component captured effects not linked to animal product restriction with five proteins (LDLR, NPY, AGRP, CD38, MFGE8) correlating positively with L and XL VLDL particles and with triglycerides (**Supplementary Fig. S11**). Studies in rats have shown that NPY controls hepatic secretion of VLDL triglycerides ^5^, suggesting that our work can also be used to understand links between metabolites and proteins independent of dietary restriction.

**Supplementary Text S5. Over-representation analysis**

The Olink Explore 1536 panel comprises four separate panels that are enriched for proteins with functions in specific biological processes: cardiometabolic, inflammation, oncology, neurology. Pathway over-representation analysis was performed for differentially abundant proteins using the online tool ShinyGO v.0.77 ^6^ and applying the total number of proteins tested (N=1,455) as background (Olink 1536 panel proteins after QC). KEGG (Release 86.1) was used as the pathway database. KEGG pathways with FDR adjusted p-value ≤ 0.05 were considered to be over-represented. We conducted over-representation analysis to test if proteins detected at altered levels were enriched for specific biological pathways. For each comparison, we tested total differentially abundant proteins for over-represented pathways, but also explored whether there were over-represented pathways when grouping proteins as up or down regulated.

Using the tested 1,455 proteins as a background, over-represented pathways were detected for:

- PR unique downregulated (N=102): metabolic pathways.
- NR unique downregulated (N=24): human papillomavirus infection, bladder cancer.
- T1 unique total (N=50): PI3K-Akt signalling pathway, focal adhesion, relaxin signalling pathway, ECM-receptor interaction, AGE-RAGE signalling pathway in diabetic complications.
- T1 unique downregulated (N=5): PI3K-Akt signalling pathway, relaxin signalling pathway, AGE-RAGE signalling pathway in diabetic complications, pathways in cancer, Ras signalling pathway, focal adhesion.

Given that the proteins assayed in the present study derive from a panel that is pre-enriched for specific functions, our analysis is underpowered and biased towards false negatives. Similarly, when applying all protein coding genes as background (19,566 HGNC genes) to interrogate over-representation of pathways, our results are biased for false positives. Therefore, this analysis is likely underpowered to provide substantial insight for the present study.

**
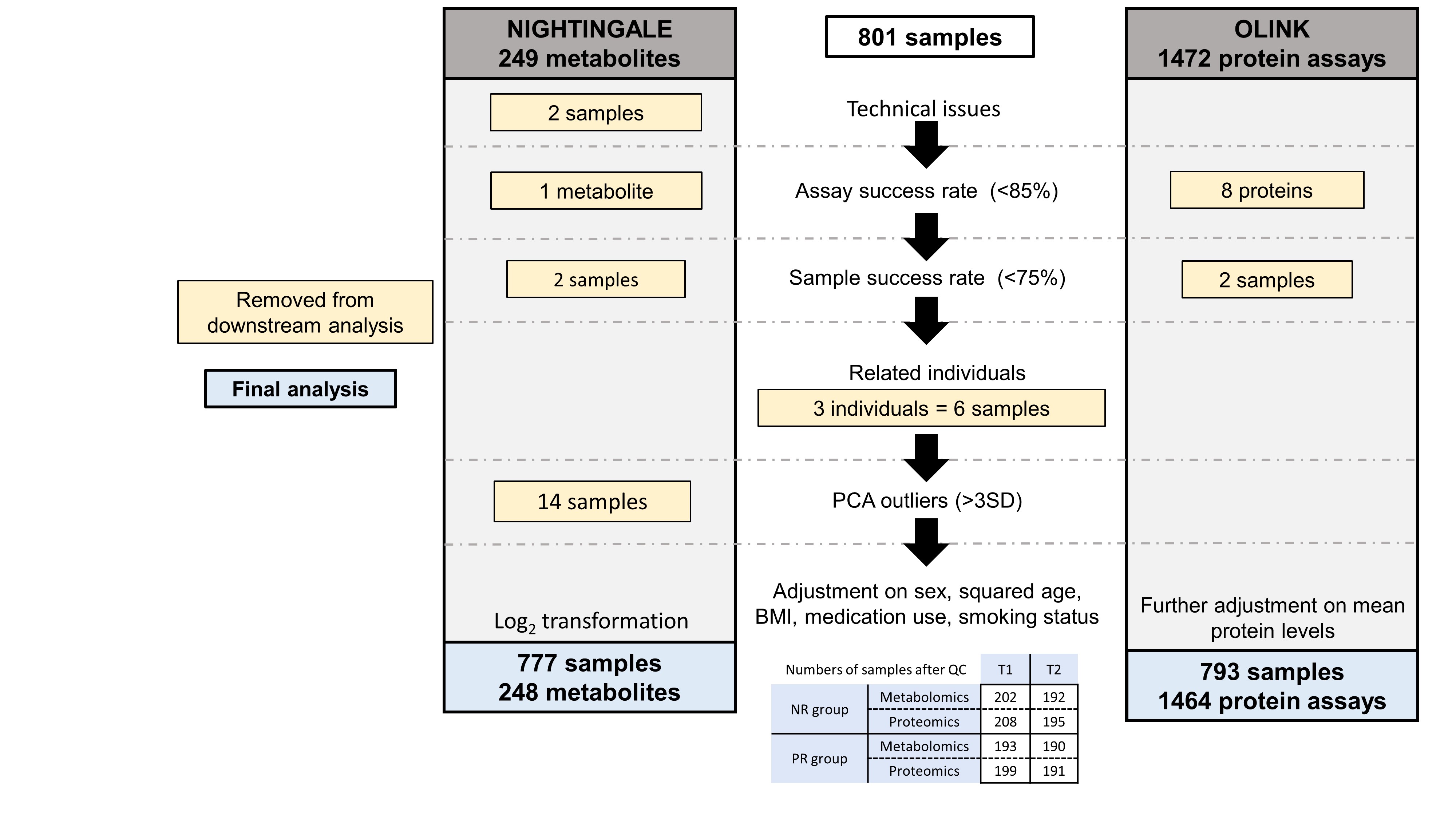
**

**Supplementary Fig. S1 Quality control flowchart for metabolomic and proteomic data.** This schema represents the quality control steps performed, showing numbers of metabolites and proteins, as well as samples removed at each step. Final numbers of metabolites and proteins and of samples in each dietary group and at each timepoint are shown in the bottom table.

**
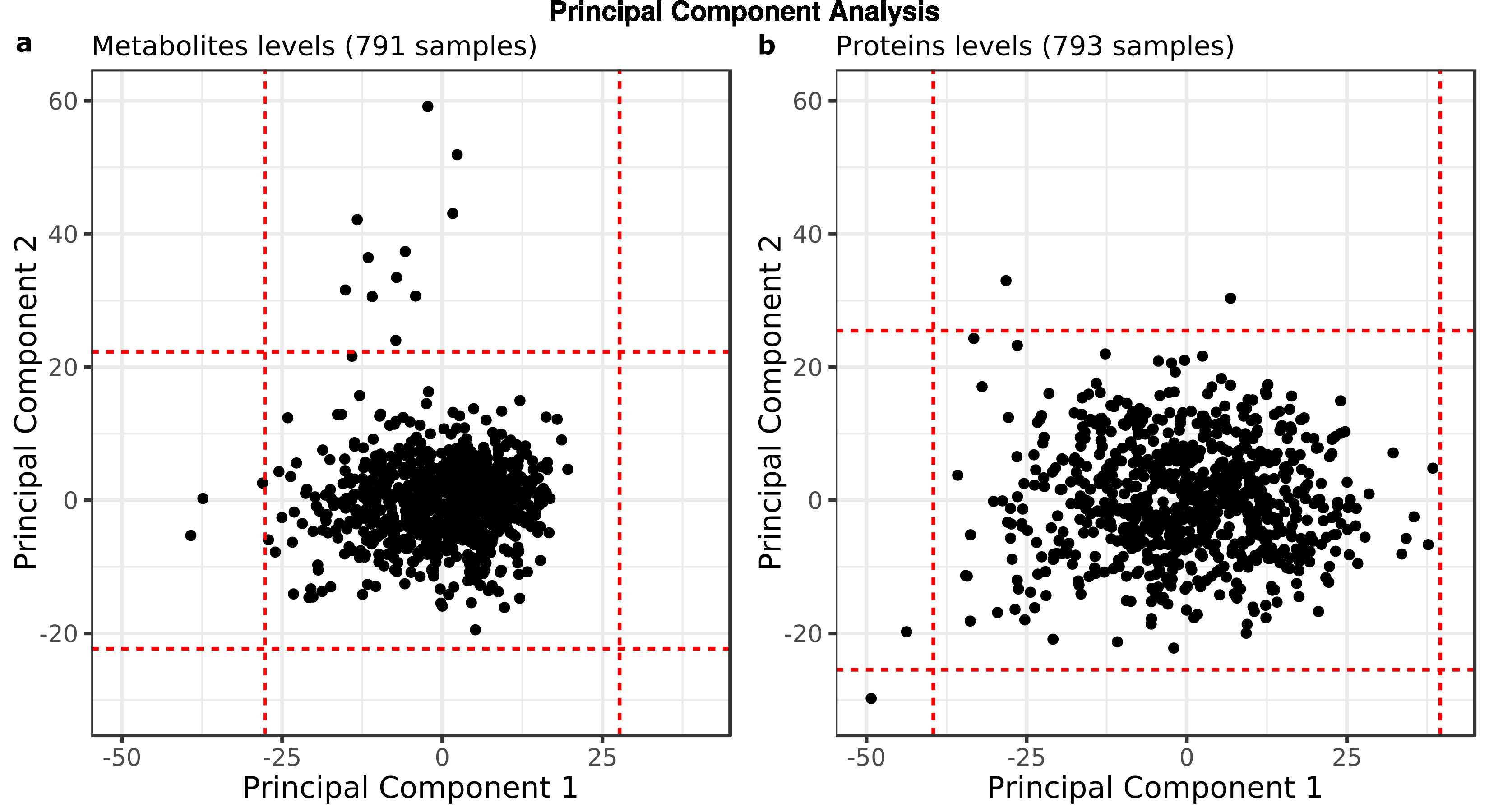
**

**Supplementary Fig. S2 Principal Component Analysis (PCA) of metabolite (a) and protein (b) levels.** PCA was performed on metabolite and protein levels before data transformation. Red lines correspond to +/- 3 standard deviations from the mean.


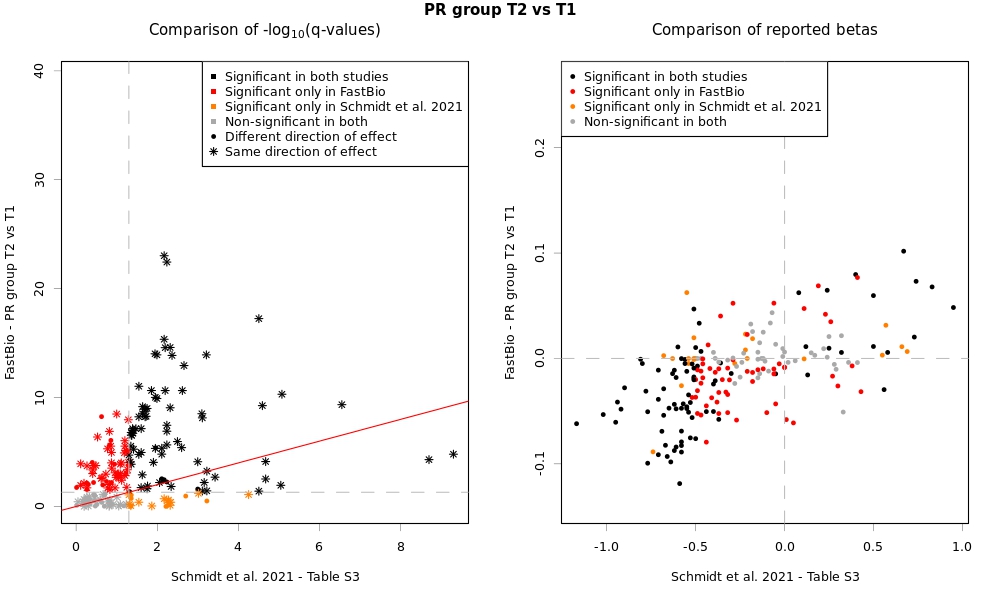


**Supplementary Fig. S3 Comparison of differentially abundant metabolites detected from T1 to T2 in the PR group, to metabolites with significantly different levels detected between vegan and meat-eating individuals.** Associations between metabolite profiles and timepoints in the PR group are compared to associations from ^7^, both in terms of p-values (panel **a**) and effect sizes (panel **b**). Metabolites are coloured according to their significance in the two studies. Star-shaped points in panel **a** represent a concordant direction of effect.


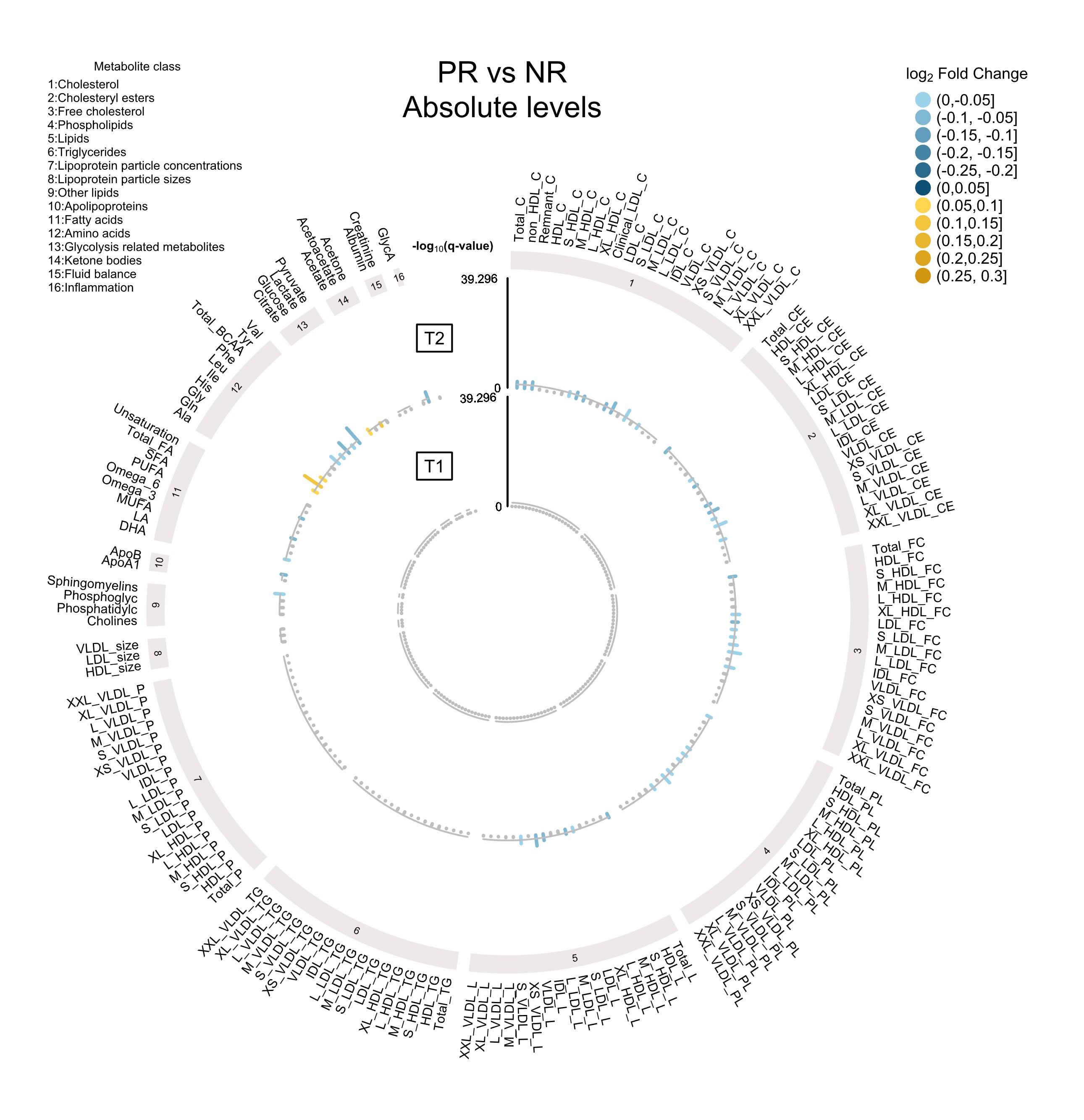


**Supplementary Fig. S4 Differentially abundant metabolites (absolute levels) detected between dietary groups at T1 and at T2.** Metabolites are grouped into classes. Within lipid classes 1-7, lipoproteins are grouped by type and are ordered by size. Differences in metabolite profiles between dietary groups are shown in the outer circle for T2 and in the inner circle for T1. The -log_10_ of the FDR-adjusted p-value (q-value) is represented in the y-axis. Yellow bars represent higher levels in the PR group whereas blue bars represent lower levels in the PR group at each timepoint. Metabolites shown in grey are not significant.

**
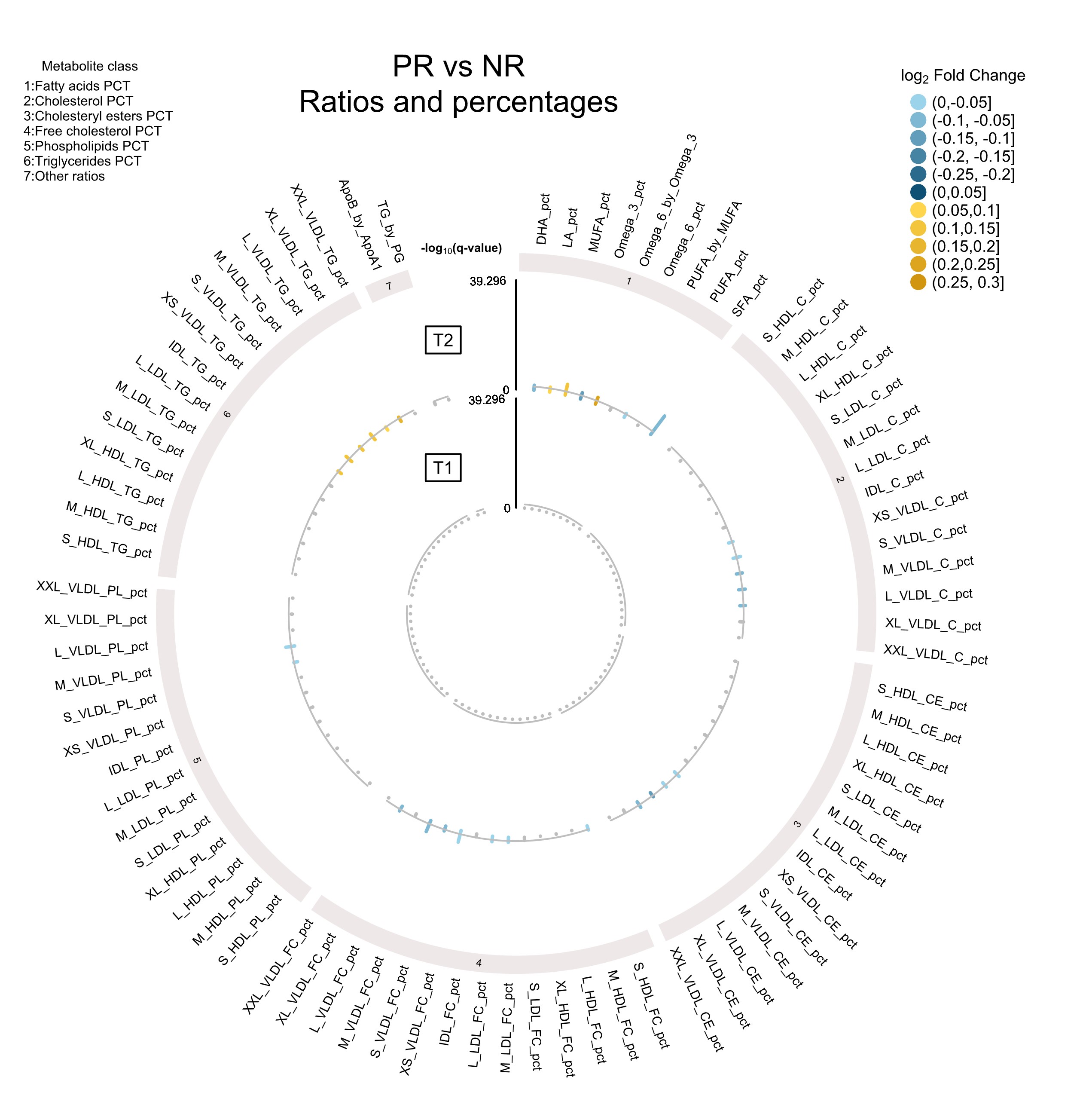
**

**Supplementary Fig. S5 Differentially abundant metabolites (ratios and percentages) detected between dietary groups at T1 and at T2.** Metabolites ratios and percentages are grouped into classes. Within each class, lipoproteins are grouped by type and are ordered by size. Differences in metabolite profiles between dietary groups are shown in the outer circle for T2 and in the inner circle for T1. The -log_10_ of the FDR-adjusted p-value (q-value) is represented in the y-axis. Yellow bars represent higher levels in the PR group whereas blue bars represent lower levels in the PR group at each timepoint. Metabolites shown in grey are not significant.


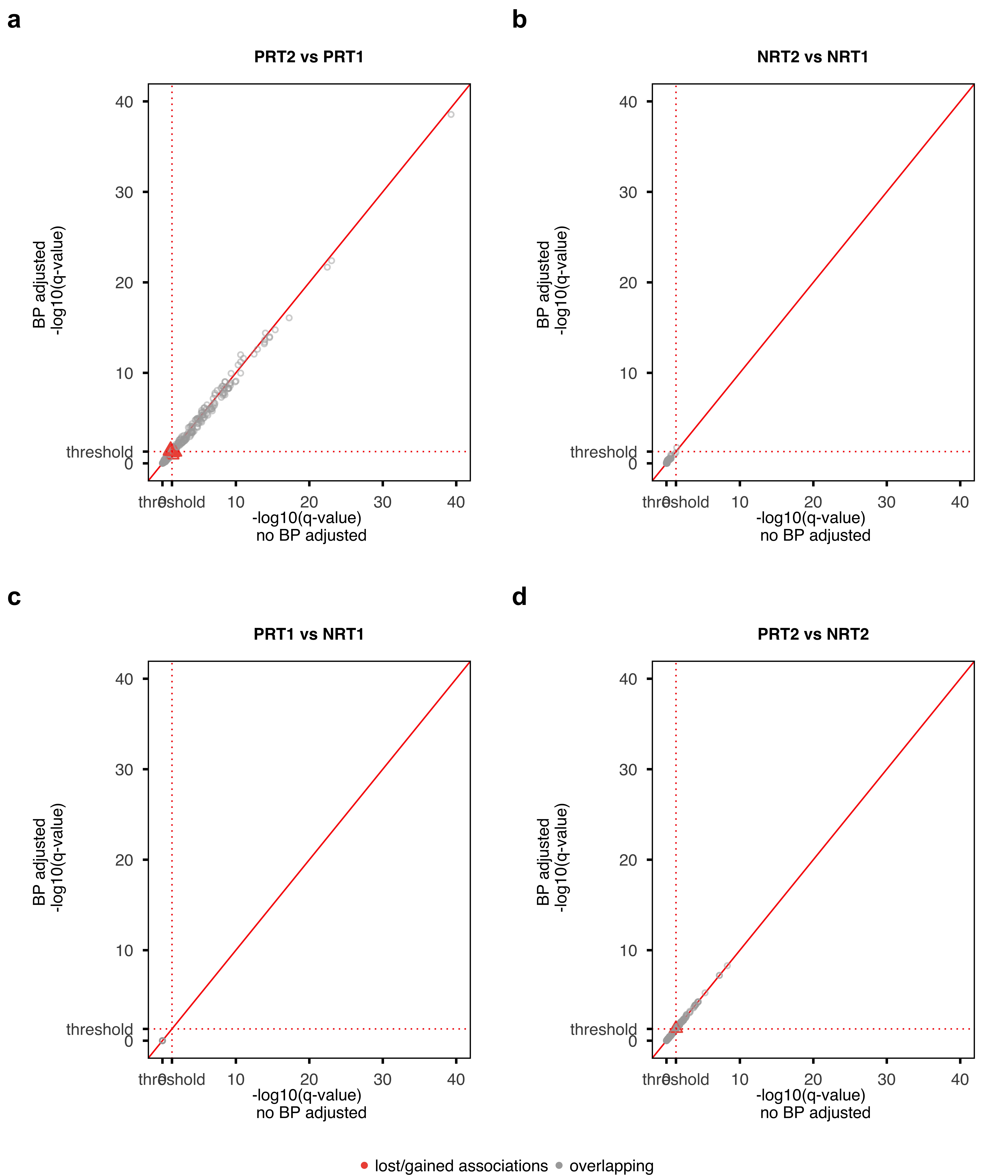


**Supplementary Fig. S6 Sensitivity analysis to explore impact of systolic and diastolic blood pressure adjustment on differentially abundant metabolites.** Sensitivity analyses with adjustment for systolic and diastolic blood pressure (SBP and DBP respectively) were conducted for all comparisons of metabolite levels (between timepoints (**a, b**) and between dietary groups (**c, d**)). -log10(q-values) from the main analysis (x axis) and from the sensitivity analysis (y axis) are shown. Associations that were lost or gained upon adjustment for SBP and DBP are shown in red. Overlapping results between the two analyses are shown in grey.


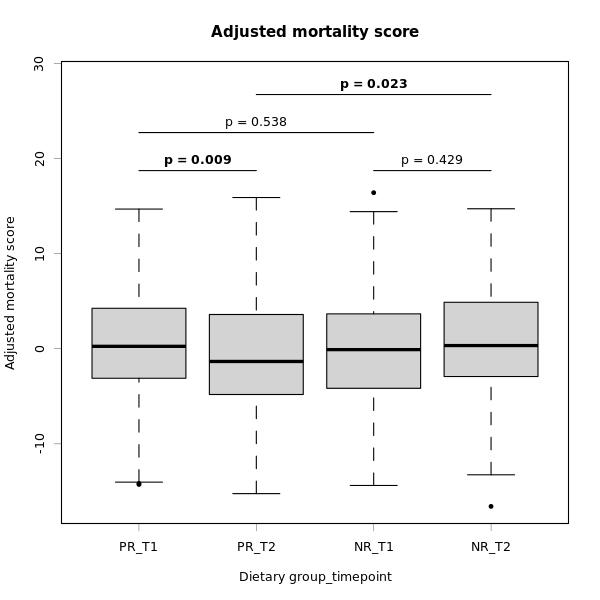


**Supplementary Fig. S7 All-cause mortality score for each dietary group at each timepoint.** The distributions of the 14-metabolite mortality score developed in ref. ^8^ are represented as boxplots and significant association p-values from a regression model of the scores against timepoints and dietary groups are shown in bold.

**
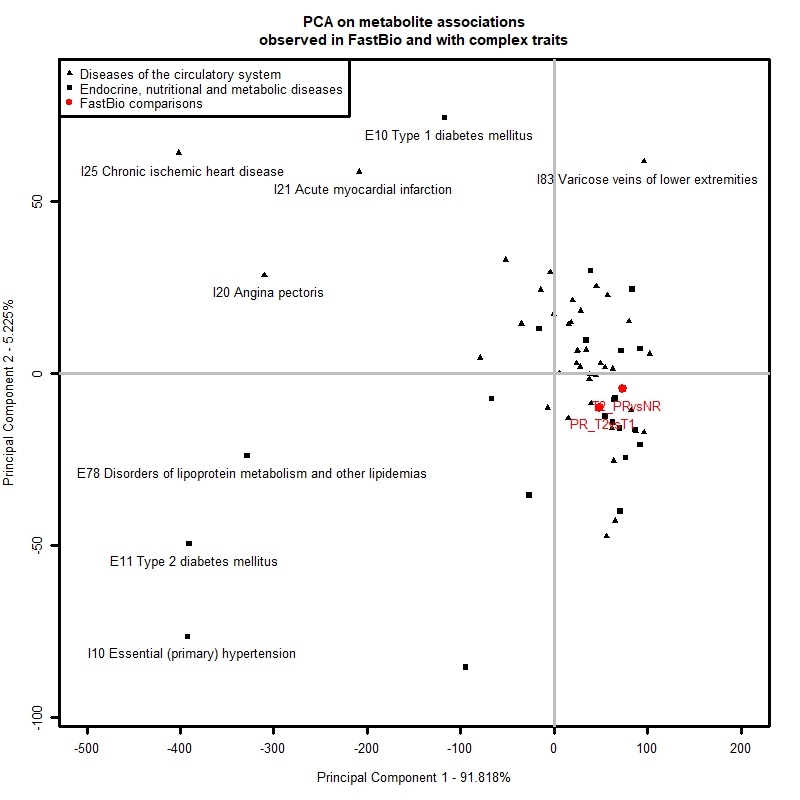
**

**Supplementary Fig. S8 Principal component analysis (PCA) of restriction-associated changes in metabolite levels to associations between metabolite levels and cardiometabolic traits from the UK Biobank.** PCA constructed on the metabolite level changes (t-statistics) described in the PR group (T2 vs T1) and differences in metabolite levels between dietary groups at T2 (PR vs NR), reported against metabolite associations linked to “diseases of the circulatory system" and “endocrine, nutritional and metabolic diseases” from the UK Biobank (ref. ^9^). In the PCA, the two first principal components are represented, and labels are reported for PR and T2 comparisons and for the diseases showing the most distinguishable pattern of associations. The PCA is mainly constructed on associations with cardiometabolic diseases and driven by those showing the strongest associations with metabolites. Other diseases, as well as PR and T2 associations, are close on the PCA as they show much lower magnitudes of associations with these metabolites. We can therefore interpret comparisons within the PR group and at T2 with diseases the furthest away, for which animal product restriction seems to be protective.

**
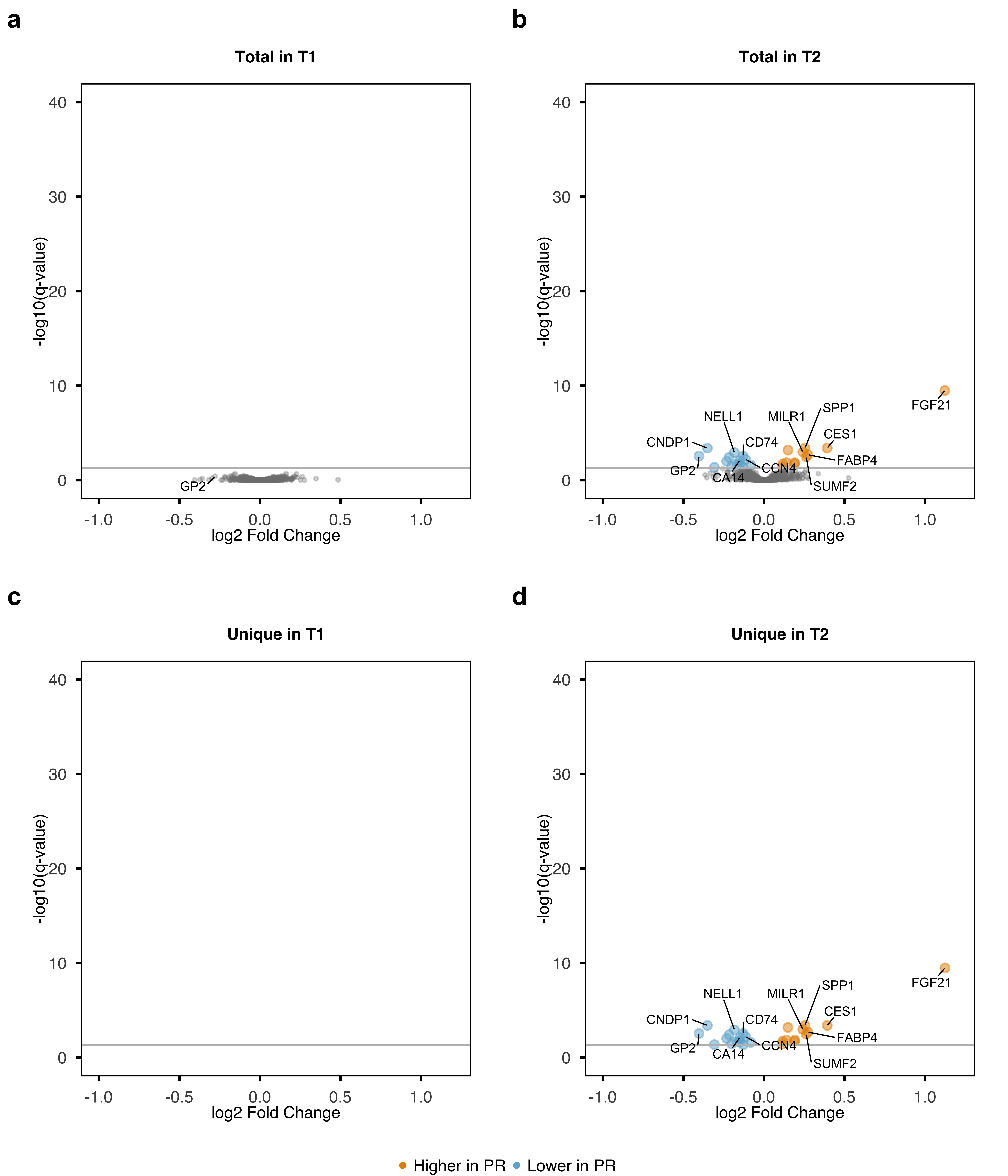
**

**Supplementary Fig. S9 Differentially abundant proteins detected between dietary groups at T1 and at T2.** Total differentially abundant proteins detected at T1 (**a**) and at T2 (**b**). Unique differentially abundant proteins detected at T1 (**c**) and at T2 (**d**). Differentially abundant proteins found in lower levels in the PR group are shown in blue whereas differentially abundant proteins found in higher levels in the PR group are shown in yellow. Proteins shown in grey are not significant.


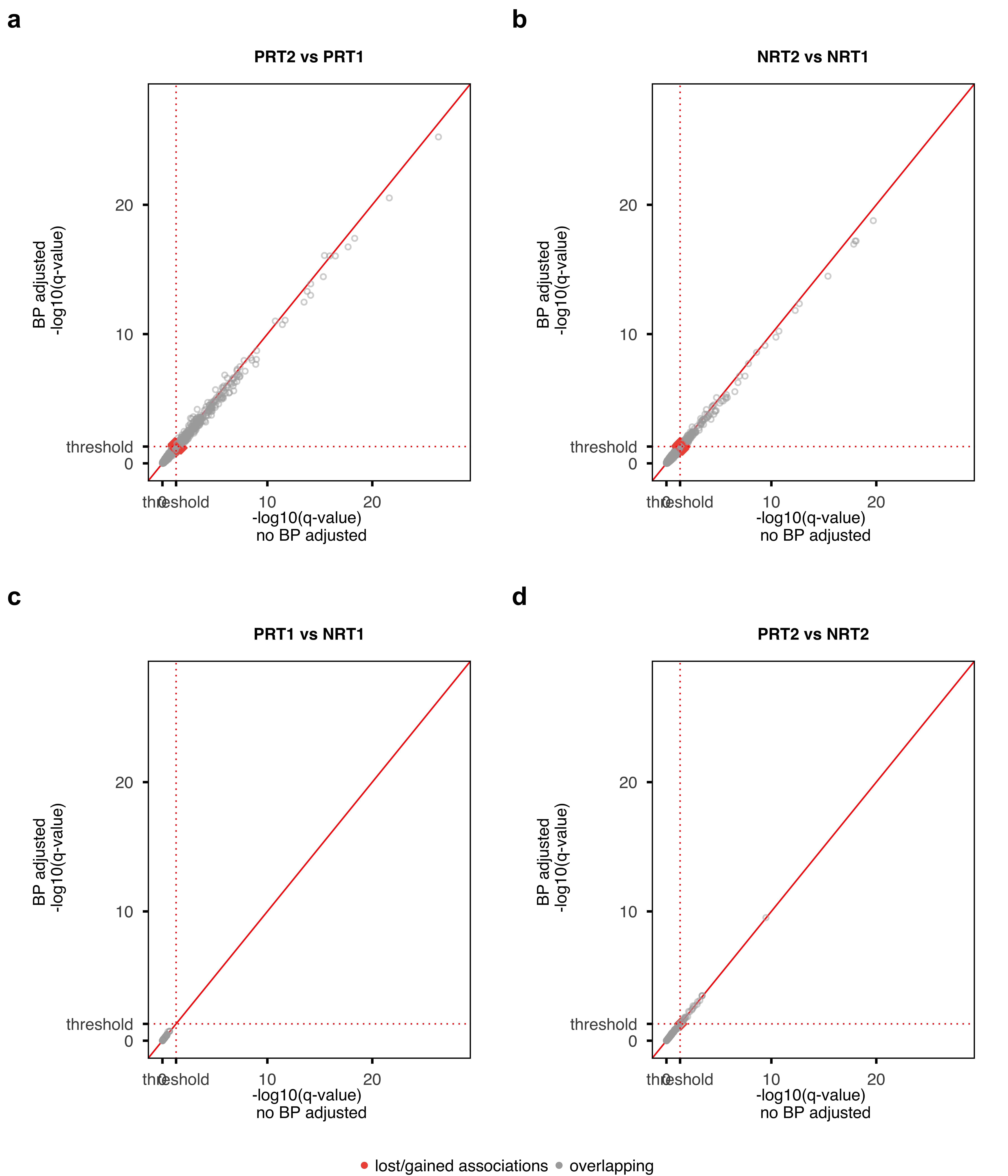


**Supplementary Fig. S10 Sensitivity analysis to explore impact of systolic and diastolic blood pressure adjustment on differentially abundant proteins.** Sensitivity analyses with adjustment for systolic and diastolic blood pressure (SBP and DBP respectively) were conducted for all comparisons of protein abundance (between timepoints (**a, b**) and between dietary groups (**c, d**)). -log10(q-values) from the main analysis (x axis) and from the sensitivity analysis (y axis) are shown. Associations that are lost or gained upon adjustment for SBP and DBP are shown in red. Overlapping results between the two analyses are shown in grey.


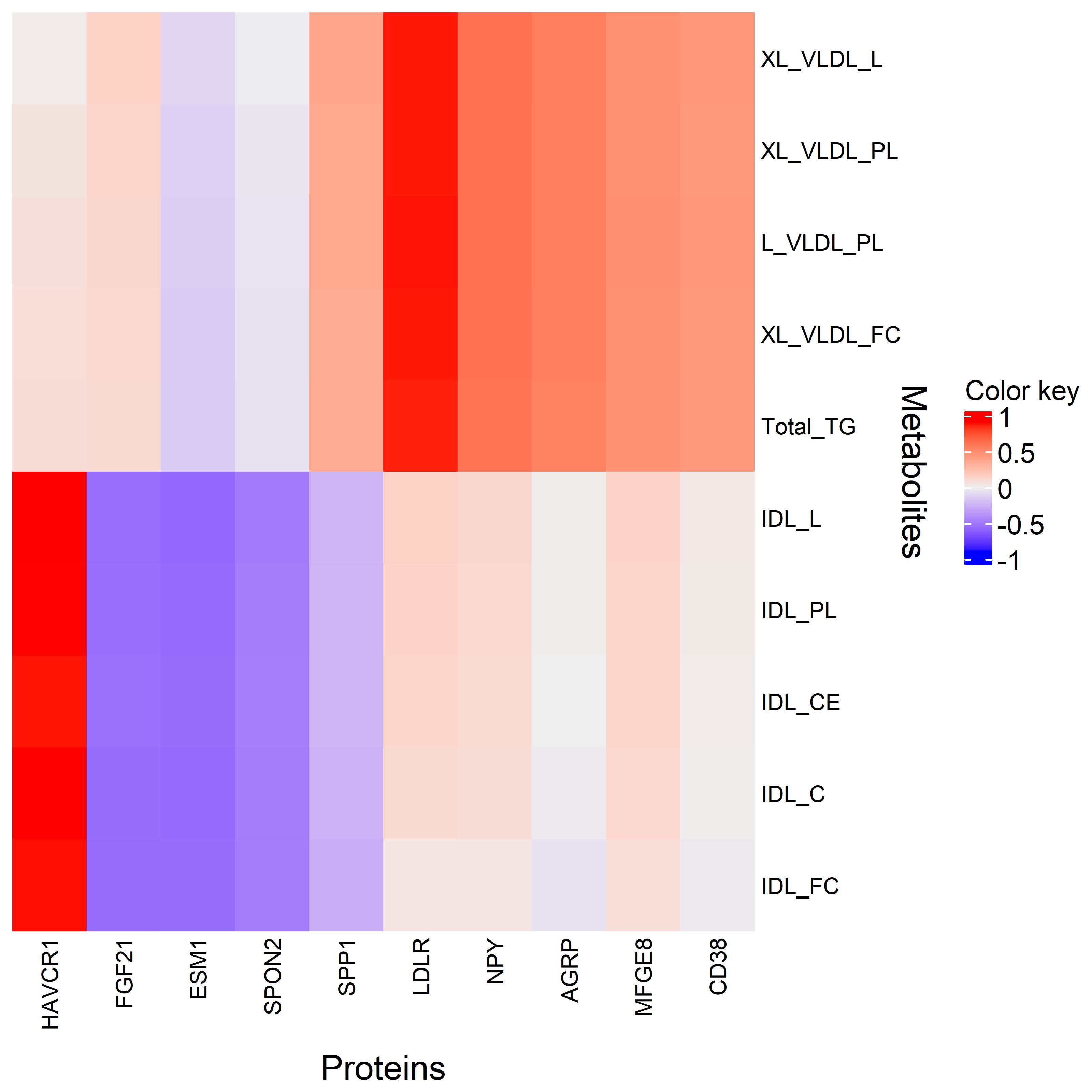


**Supplementary Fig. S11** **Metabolite-protein correlations.** Correlations between optimally selected proteins (x axis) and metabolites (y axis) by the sPLS method in mixOmics.

**
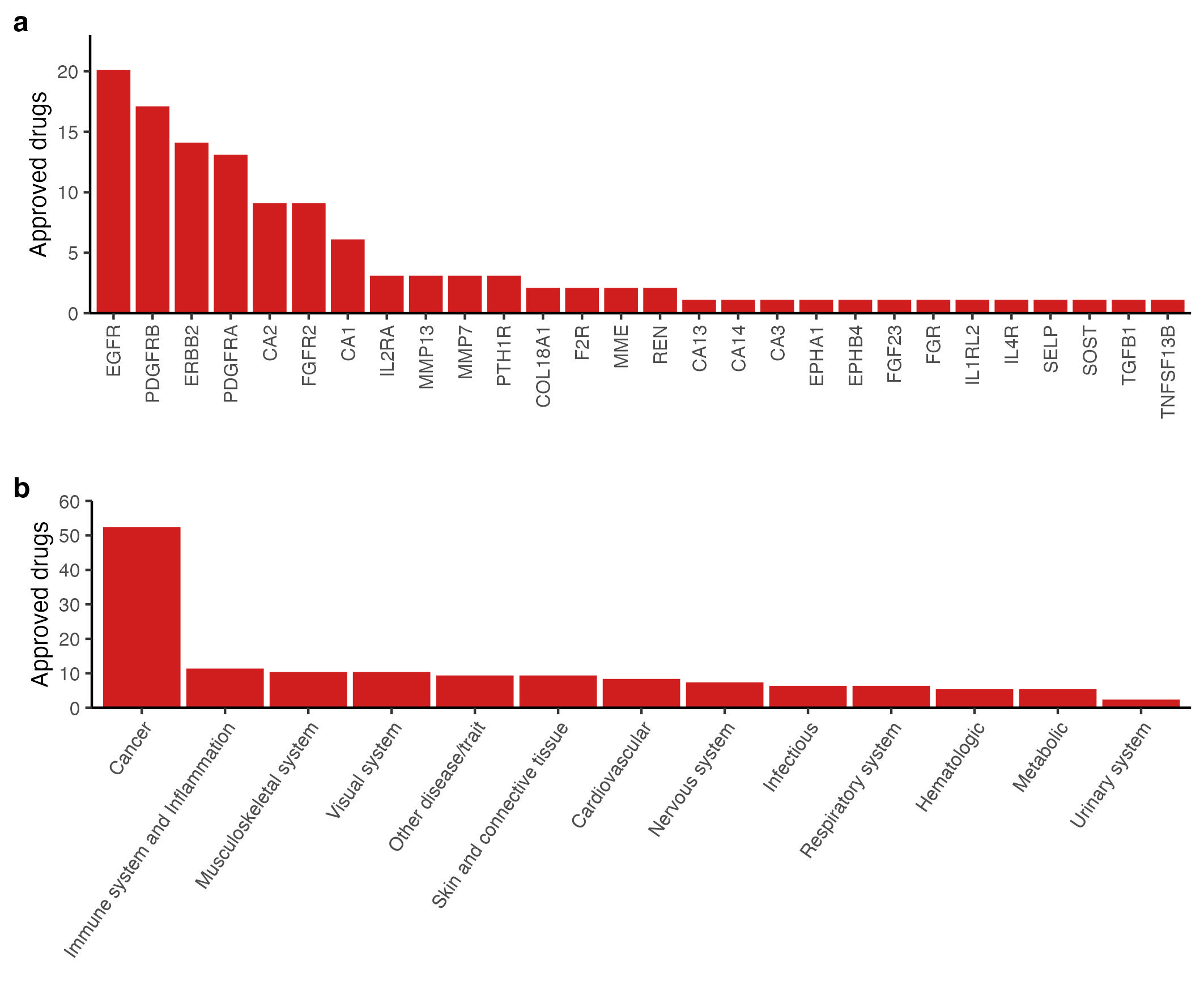
**

**Supplementary Fig. S12 PR-unique differentially abundant proteins as targets of approved drugs and associated disease indications.** **a**, Number of approved drugs (excluding withdrawn drugs) that target differentially abundant proteins detected only in the PR group. **b**, Number of approved drugs (from above) per disease indication.


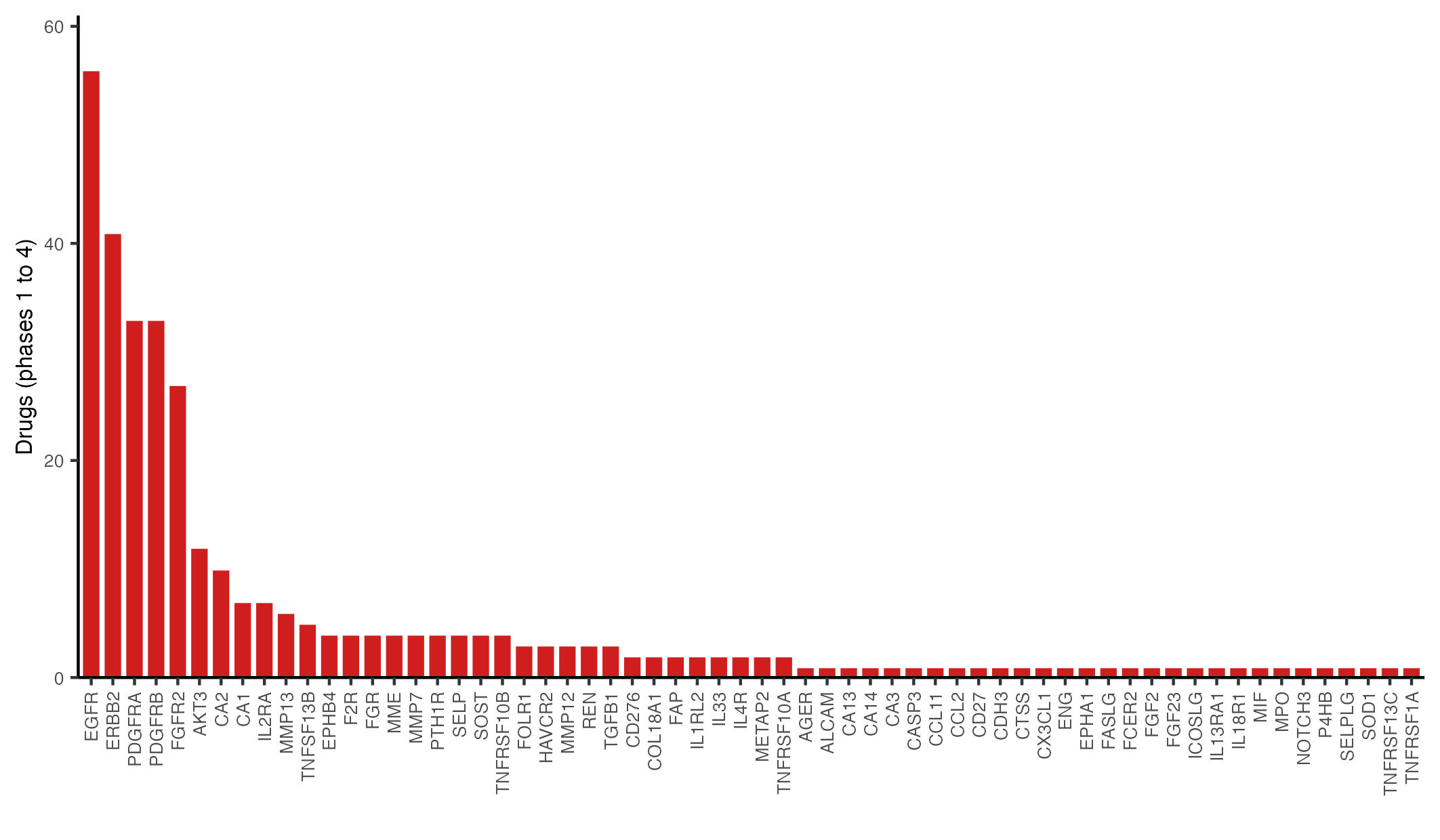


**Supplementary Fig. S13 PR-unique differentially abundant proteins as targets of phase 1-4 drugs.** Number of phase 1-4 drugs (excluding clinical trials with withdrawn, unknown or terminated status) that target differentially abundant proteins detected only in PR individuals.

**
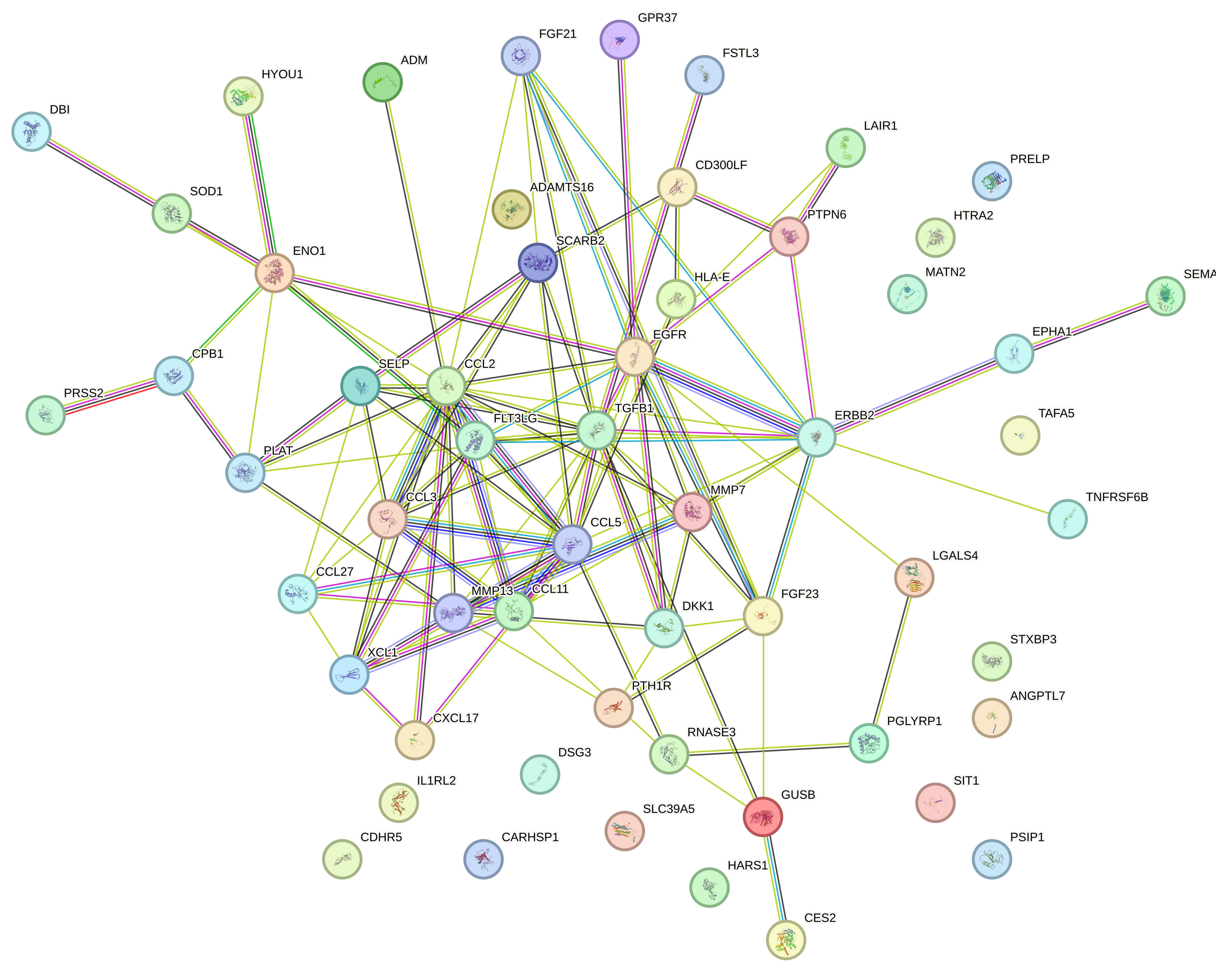
**

**
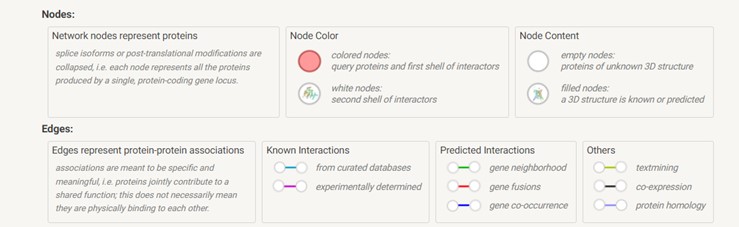
**

**Supplementary Fig. S14.** **Interaction network of 55 proteins with suggestive links to FGF21 signalling in humans.** The nodes represent proteins, and edges represent protein-protein interactions.


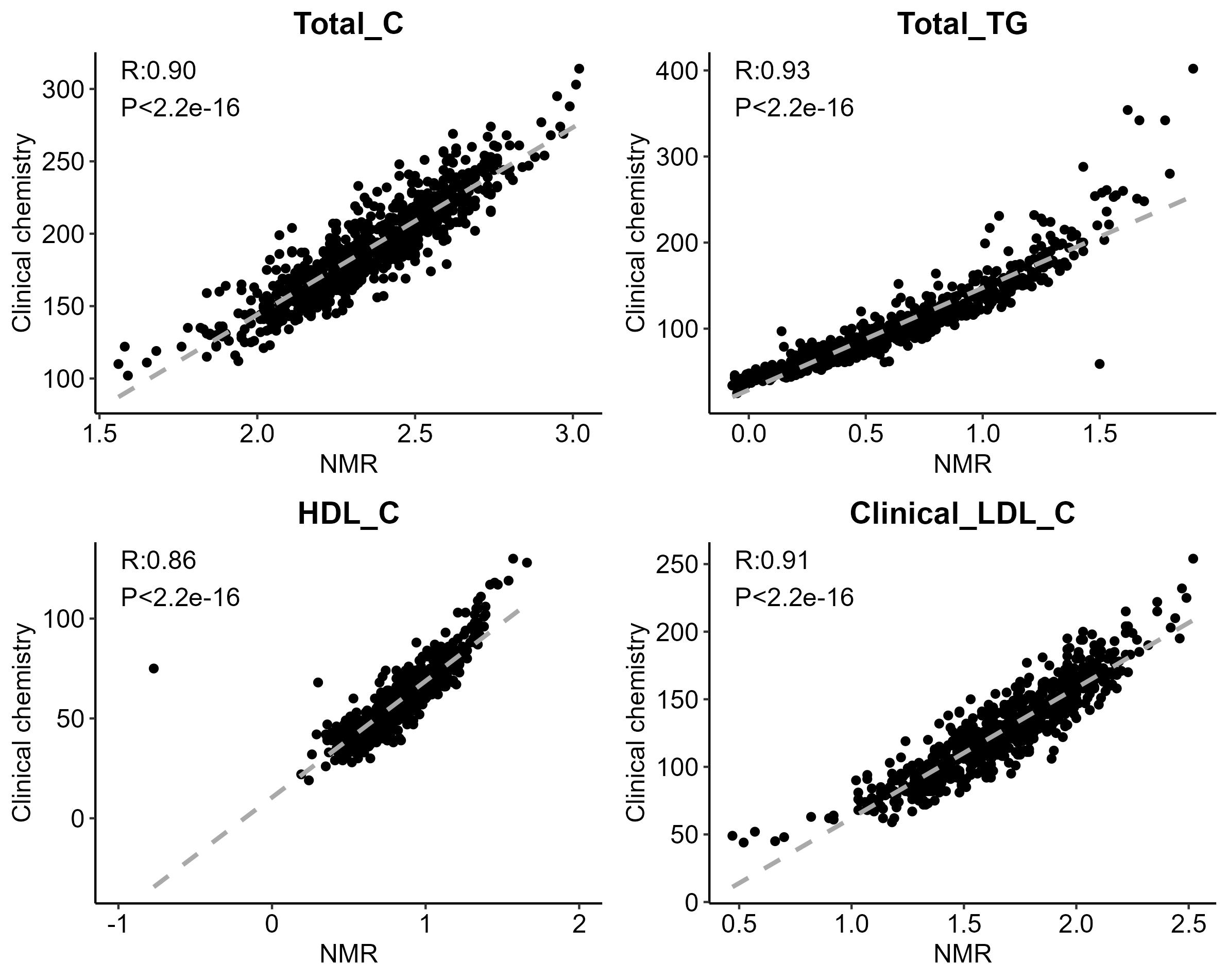


**Supplementary Fig. S15** **Comparisons of Nightingale Health NMR biomarker measurements to routine clinical chemistry measurements.** Scatterplots of total cholesterol, total triglycerides, HLD cholesterol and clinical LDL cholesterol, for which both NMR and clinical chemistry measurements are available in FastBio participants ^10^. Correlation coefficients (R) represent linear Pearson’s correlations and dashed line represents the regression line. Clinical chemistry was carried out at the Central Laboratories of the Interbalkan Hospital of Thessaloniki, Greece (cobas c 311 analyzer, Roche Diagnostics). ‘Clinical LDL cholesterol’ is the measure that provides concentrations consistent with routine clinical chemistry and the Friedewald equation for LDL- cholesterol. It is calibrated to match standard laboratory assays.


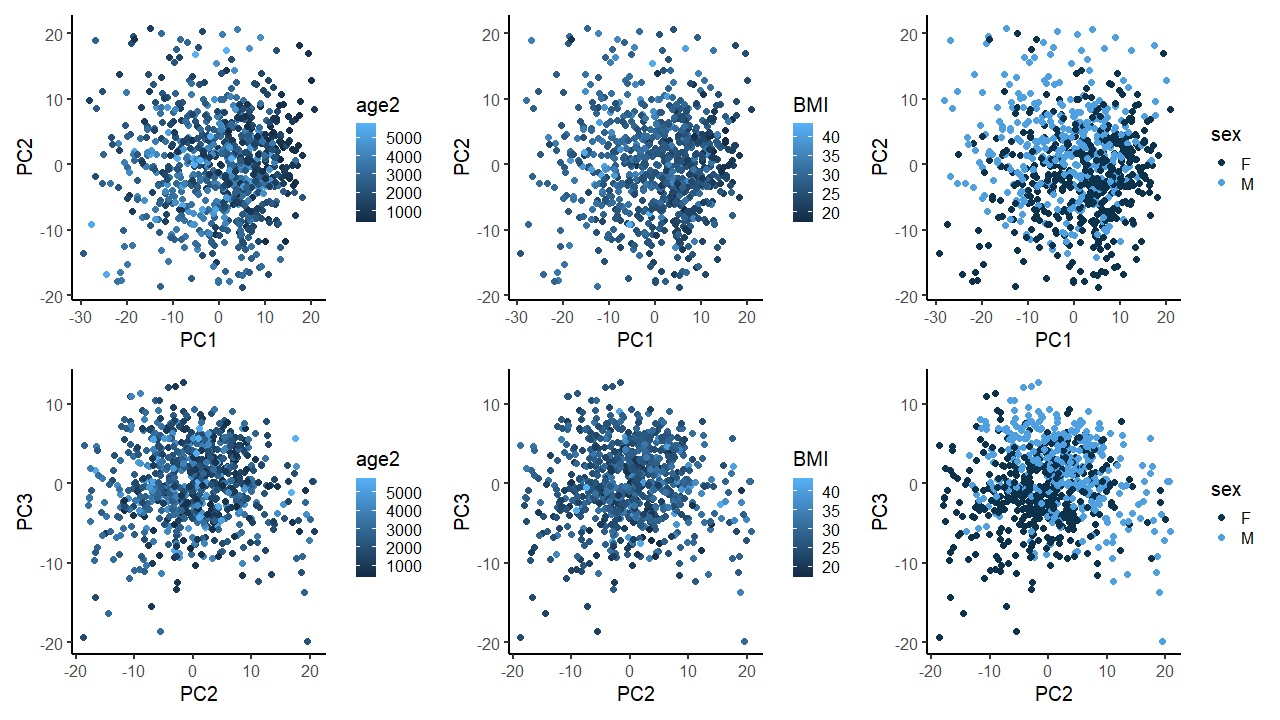


**Supplementary Fig. S16 Principal component plot on metabolite levels.** Scatterplots of PC1 vs PC2; PC2 vs PC3 based on metabolite levels colored by age^2^, BMI (kg/m^2^) and sex.


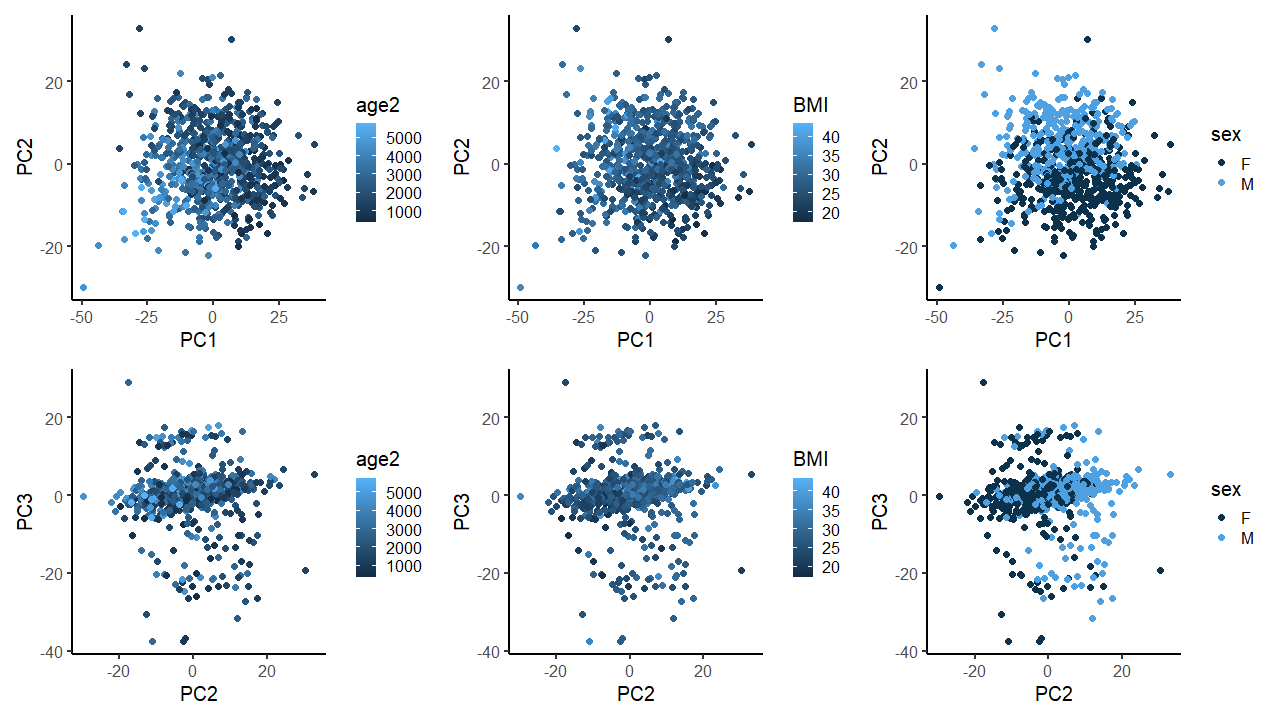


**Supplementary Fig. S17 Principal component plot on protein levels.** Scatterplots of PC1 vs PC2; PC2 vs PC3 based on protein levels colored by age^2^, BMI (kg/m^2^) and sex.

**Supplementary Data 1. Sociodemographic traits and geographic origin of study participants.** (xlsx)

**Supplementary Data 2. Overview of differentially abundant metabolites and differentially abundant proteins between timepoints and dietary groups.** (xlsx)

**Supplementary Data 3. Phase 1-4 drugs targeting PR-unique proteins and associated conditions.** (xlsx)

**Supplementary Data 4. Phase 1-4 clinical trials for proteins displaying the greatest magnitude of animal product restriction-associated changes.** (xlsx)

**Supplementary Data 5. Proteins with suggestive links to FGF21**. **Upon adjustment for FGF21 levels, 55 out of the 411 PR-detected differentially abundant proteins were no longer significant.** (xlsx)

**Supplementary Data 6. Mendelian randomization analysis.** (xlsx)

**References**

1. Sarri, K.O., Linardakis, M.K., Bervanaki, F.N., Tzanakis, N.E. & Kafatos, A.G. Greek Orthodox fasting rituals: a hidden characteristic of the Mediterranean diet of Crete. *Br J Nutr* **92**, 277-284 (2004).

2. Georgakouli, K. *et al.* The Effects of Greek Orthodox Christian Fasting during Holy Week on Body Composition and Cardiometabolic Parameters in Overweight Adults. *Diseases* **10** (2022).

3. Le Cao, K.A., Rossouw, D., Robert-Granie, C. & Besse, P. A sparse PLS for variable selection when integrating omics data. *Stat Appl Genet Mol Biol* **7**, Article 35 (2008).

4. Rohart, F., Gautier, B., Singh, A. & Le Cao, K.A. mixOmics: An R package for 'omics feature selection and multiple data integration. *PLoS Comput Biol* **13**, e1005752 (2017).

5. Bruinstroop, E. *et al.* Hypothalamic neuropeptide Y (NPY) controls hepatic VLDL-triglyceride secretion in rats via the sympathetic nervous system. *Diabetes* **61**, 1043-1050 (2012).

6. Ge, S.X., Jung, D. & Yao, R. ShinyGO: a graphical gene-set enrichment tool for animals and plants. *Bioinformatics* **36**, 2628-2629 (2020).

7. Schmidt, J.A. *et al.* NMR Metabolite Profiles in Male Meat-Eaters, Fish-Eaters, Vegetarians and Vegans, and Comparison with MS Metabolite Profiles. *Metabolites* **11** (2021).

8. Deelen, J. *et al.* A metabolic profile of all-cause mortality risk identified in an observational study of 44,168 individuals. *Nat Commun* **10** (2019).

9. Julkunen, H. *et al.* Atlas of plasma NMR biomarkers for health and disease in 118,461 individuals from the UK Biobank. *Nat Commun* **14**, 604 (2023).

10. Loizidou E. M., S.A., Barbounakis P., Glentis S., Dimopoulos A., Anezaki M., Rouskas P., Kontoyiannis I., Demiris N., Scarmeas N., Yannakoulia M., Rouskas K., Dimas A.S. Effects of short-term restriction of animal products on blood biomarkers and cardiovascular disease risk. Preprint at <https://www.medrxiv.org/content/10.1101/2023.05.17.23290094v5> (2024).
